# Genome-wide H3K4me3 profiling of circulating immune cells reveals dynamic epigenetic reprogramming during acute critical COVID-19

**DOI:** 10.3389/fimmu.2026.1907275

**Published:** 2026-08-20

**Authors:** Jan Niklas Thon, Judith Schenz, Mascha Onida Fiedler, Uta Merle, Monica Boxberger, Florian Uhle, Markus Alexander Weigand, Dominik Schaack, Benedikt Hermann Siegler

**Affiliations:** 1 Medical Faculty Heidelberg, Department of Anesthesiology, Heidelberg University, Heidelberg, Baden-Württemberg, Germany; 2 Medical Faculty Heidelberg, Department of Gastroenterology and Infectious Diseases, Heidelberg University, Heidelberg, Baden-Württemberg, Germany; 3 University Center for Acute Respiratory Distress Syndrome (ARDS) and Weaning, Heidelberg University Hospital, Heidelberg, Baden-Württemberg, Germany

**Keywords:** COVID-19, epigenetics, H3K4me3, histone modifications, neutrophil hyperactivation, SARS-CoV-2, sepsis

## Abstract

**Introduction:**

Severe COVID-19 is associated with innate immune dysregulation resembling sepsis-induced immunoparalysis. Epigenetic mechanisms, particularly changes in H3K4me3 enrichment at gene promoters, have been observed in immune tolerance and monocyte dysfunction in sepsis. Whether comparable H3K4me3 alterations occur during acute critical COVID-19 illness has not been investigated.

**Methods:**

In this prospective single-center study, 46 hospitalized COVID-19 patients were enrolled, of whom 27 were treated in the intensive care unit (ICU group) and 19 on the normal ward (non-ICU group). Genome-wide H3K4me3 ChIP-seq was performed on PBMCs at hospital admission (T1) in the total cohort and after seven days (T2) in the ICU group. Monocyte HLA-DR expression and *ex vivo* TLR-stimulated cytokine secretion were assessed as functional immune readouts.

**Results:**

Among 706 differentially bound consensus peaks with promoter association between ICU and non-ICU groups, 704 showed increased H3K4me3 occupancy in ICU patients, predominantly at neutrophil effector gene loci, supported by pathway enrichment of neutrophil degranulation and innate immune activation. Monocyte HLA-DR expression and *ex vivo* TLR-stimulated IL-6 secretion were persistently reduced throughout the first week of ICU treatment. Longitudinal profiling in the ICU group revealed a shift from an interferon-driven chromatin signature at admission toward sustained innate immune activation and ECM remodeling at day seven.

**Conclusion:**

This study provides the first genome-wide H3K4me3 characterization of circulating immune cells during acute critical COVID-19, demonstrating that epigenetic reprogramming is an active and dynamic process that mirrors the functional immune dysregulation observed in these patients.

## 1 Introduction

It has been more than half a decade since Chinese authorities reported an increased number of pneumonia cases caused by an unknown Coronavirus in December 2019. The virus later known as severe acute respiratory syndrome (SARS) Coronavirus-19 (SARS-CoV-2) caused one of the most significant global health threats in the 21^st^ century (1). Most infected patients show no or only mild respiratory symptoms, and with the advent of better therapeutic options and vaccine-mediated immunity initially reported severe disease progression of ~23% and mortality rates of 5.6% could be reduced significantly in the following years (2–5). While overall mortality was reduced, mortality among infected critically ill patients admitted to the ICU remains high, especially in patients in need of mechanical ventilation, despite therapeutic advancement and better knowledge of the disease (6–8).

Mortality rates of critically ill COVID-19 patients are comparable with those from patients with bacterial sepsis (9). According to the third international consensus definition, sepsis is described as a life-threatening organ dysfunction caused by a dysregulated immune response to pathogens (10). This definition matches the disease progression of many critically ill patients infected with SARS-CoV-2, who develop acute respiratory distress syndrome (ARDS) and multi-organ dysfunction, considering COVID-19 a viral sepsis (11–13). In sepsis, a generalized inflammatory reaction is accompanied by a vast release of proinflammatory cytokines and simultaneously by high levels of anti-inflammatory cytokines trying to compensate the excessive immune response (14, 15). Critically ill patients with COVID-19 also show elevated levels of both pro-inflammatory (e.g., IL-1ß, IL-6, IL-12 and TNF-α) and anti-inflammatory cytokines (e.g., IL-4 and IL-10) (16, 17). It is known that the cytokine profile in critically ill patients with bacterial sepsis can be used as a predictor of disease severity (18). A meta-analysis by Dhar et al. was able to show similar correlations between IL-6 and IL-10 levels and disease severity for patients with COVID-19 (19). Additionally, in severe COVID-19 cases, neutrophil hyperactivation has been identified as a central mechanism of immune dysfunction, leading to an exaggerated immune response including oxidative burst, degranulation and the formation of neutrophil extracellular traps (NETs) by NETosis (20). The release of proteolytic enzymes, like neutrophil elastase (ELANE) as well as matrix metalloproteinase (MMP) 8 and 9, contribute to extracellular matrix remodeling, alveolar barrier disruption, and lung tissue damage, and higher plasma levels correlate with disease severity (21–23).

Regarding antigen presentation in critically ill COVID-19 patients, both pro-inflammatory cytokines, particularly IL-6, and neutrophil elastase can decrease human leucocyte antigen DR (HLA-DR) expression on the surface of antigen presenting cells, which is also associated with disease severity (24–26). HLA-DR is an established predictor for mortality in sepsis and septic shock as well as a surrogate for immunosuppression (27, 28). Just like in sepsis, HLA-DR expression is reduced in patients with SARS-CoV-2 (29). Low levels of HLA-DR expression are associated with monocyte anergy and an insufficient antigen presentation (30).

In previous work, we could demonstrate that diminished HLA-DR expression on monocyte surface and reduced transcription of major histocompatibility complex class II (MHC-II) proteins in patients with sepsis was accompanied by specific alterations of the chromatin landscape and a differential binding of the epigenetic regulator CCCTC-binding factor (CTCF) within the MHC-II region (31–33). Epigenetic changes seem to play an important role in the immune response of patients with COVID-19 as well. Of note is the trimethylation of histone H3 on lysine 4 (H3K4me3), which is an activating chromatin mark, highly enriched at promoters of transcriptionally active genes, and associated with increased gene expression (34, 35). In the context of innate immune tolerance, there is a link between selectively reprogramming of the chromatin landscape of monocytes and macrophages and repeated endotoxin exposure, with reduced H3K4me3 enrichment at promoter regions of pro-inflammatory genes upon secondary challenge while antimicrobial effector genes retain the activating chromatin mark (36). While such activating marks are linked to reduced responsiveness in immune tolerance, H3K4me3 is equally a hallmark of innate immune priming and trained immunity, where it primes immune effector genes for enhanced expression upon subsequent stimulation (37). Trained immunity signatures have been described in individuals convalescing from COVID-19 (38).

The mechanism of epigenetic silencing has been directly implicated in patients with bacterial sepsis-associated immune dysfunction, as ChIP-seq analysis of monocytes showed a reduction in H3K4me3 and H3K9ac occupancy at differential MHC-II loci including the class II major histocompatibility complex transactivator (CIITA), connecting a reduction in H3K4me3 binding to impaired HLA-DR expression (31). Consistent with this, a recent integrative transcriptomic analysis of circulating immune cells from critical COVID-19 patients identified a regulatory network predictive of a specific metabolic and epigenetic imprint, pointing to epigenetic reprogramming as a feature of severe disease (39). Recent evidence further suggests that severe COVID-19 induces long lasting epigenomic reprogramming of hematopoietic stem and progenitor cells, with changes in progeny circulating innate immune cells persisting for up to one year (40). Of note, such epigenetic alterations and the functional immune phenotype are not necessarily linked through the same gene loci, and differences in cytokine responsiveness may arise independently of differential histone modifications at the corresponding cytokine genes.

Whether comparable H3K4me3 alterations occur during acute critical COVID-19 illness, and whether these changes reflect the functional changes in the immune system observed in these patients, has not yet been investigated. Therefore, in this present study, we characterized the genome-wide H3K4me3 landscape of circulating immune cells in critically ill COVID-19 patients admitted to the ICU in comparison to patients with moderate disease course. Additionally, we assessed longitudinal changes over the course of the ICU treatment.

## 2 Methods

### 2.1 Ethics and patients

All experiments were approved by the ethics committees of the medical faculty of Heidelberg University (Alte Glockengießerei 11/1, 69115, Heidelberg, Germany, trial code: S-176/2020) were compliant with the principles stated in the Declaration of Helsinki and its later amendments, and registered in the German Clinical Trial Register (Trial number: DRKS00327189). Patient’s written consent was obtained in advance, except in cases of incapacity, in which consent was obtained from legal representatives.

In this prospective, observational study, patients admitted to Heidelberg University Hospital due to SARS-CoV-2 infection between December 29^th^, 2020, and April 27^th^, 2021, were screened for eligibility to participate. Participants had to be at least 18 years of age and had to be admitted to the hospital no longer than 48h before study inclusion. General exclusion criteria were enrolment in an interventional study, pre-existing immunosuppression, chronic viral infections (e.g. HIV, HCV, HBC), pregnancy, or anemia. Patient allocation to the ICU or normal ward was performed according to hospital standard operating procedure. Individuals had to meet the following criteria to be administered to the hospital (1): C-reactive protein (CRP) ≥ 20 mg/dl (2); Ferritin > 400 mg/dl (3); abnormal white blood cell count (4); peripheral oxygen saturation < 95%. Patients with a peripheral oxygen saturation ≤ 93% on room air as well as patients with supplemental oxygen of at least 4 L/min were administered to the ICU. Patients with a peripheral oxygen saturation ≥ 94% on room air and no hypotension were administered to the normal ward (`non-ICU group´). None of the patients received vaccination against SARS-CoV-2 prior to enrolment. Blood samples were drawn within 24 h after hospitalization (T1), in both groups, and after 7 days (T2), only in the ICU group, and were processed immediately.

### 2.2 Data collection and standard laboratory parameters

An assessment of socio-demographic and clinical features, including pre-existing comorbidities based on the Charlson comorbidity index (41), was conducted, as well as an analysis of standard laboratory parameters, which were measured in the routine hospital laboratory according to in-house standards.

### 2.3 Cell isolation

For peripheral blood mononuclear cell (PBMC) separation, density gradient centrifugation was used with Leucosep tubes prefilled with separation medium (greiner bio-one, Kremsmuenster, Austria). Following centrifugation, the PBMC layer was aspirated and washed with ice-cold isolation buffer (phosphate-buffered saline supplemented with 2 mM ethylenediaminetetraacetic acid (Thermo Fisher Scientific Inc, Waltham, USA) and 0.5% protease-free bovine serum albumin (Carl Roth, Karlsruhe, Germany)). To perform a hypotonic lysis to remove remaining erythrocytes, cells were resuspended in 20 mL 0.2% sodium chloride (Merck KGaA, Darmstadt, Germany) for 20 seconds before 20 mL of 1.6% sodium chloride was added for restoration of isotonic conditions. Subsequently, cells were centrifuged), washed with ice-cold isolation buffer. The isolated PBMCs were counted using a Scepter™ 2.0 Cell Counter (Merck Millipore, Burlington, USA) that operates via impedance-based particle detection.

### 2.4 PBMC stimulation

To determine the functionality of the isolated PBMCs, 1.5 x 10^5^ cells were incubated for 24 hours with ultrapure lipopolysaccharide (LPS), Zymosan or Flagellin (all InvivoGen, San Diego, CA, USA). After incubation, cells were centrifuged, supernatant was recovered and a subsequent enzyme-linked immunosorbent assay (ELISA) was performed.

### 2.5 Enzyme-linked immunosorbent assay

In accordance with manufacturer’s guidelines, plasma derived from *ex vivo* stimulated as well as untreated samples were used for ELISA experiments to measure concentrations of the pro-inflammatory cytokine IL-6 (R&D Technologies, North Kingstown, USA).

### 2.6 Flow cytometry and monocyte surface density of HLA-DR molecules

Density of HLA-DR on monocyte cell surface was quantified by flow cytometry (FACSVerse™ flow cytometer, BD Bioscience, Heidelberg, Germany). EDTA-anti-coagulated whole blood (50 µl) was incubated with 20 µl anti-HLA-DR antibody (Quantibrite anti-HLA-DR/Monocyte antibody, BD Bioscience, Heidelberg, Germany) before lysis of erythrocytes (FACS Lysing solution BD Bioscience, Heidelberg, Germany) was performed as previously described and according to manufacturer`s instructions (42). HLA-DR receptor density was calculated based on measured median fluorescence intensity (MFI) once monocytes had been gated using CD14 expression. The density of HLA-DR molecules on cell surfaces (`counts per monocyte´) was quantified by conversion of the determined MFI values using daily measured four-point calibration curves (Quantibrite PE Beads, BD Bioscience, Heidelberg, Germany).

For the analysis of myeloid-derived suppressor cell (MDSC) subsets, 1.5 x 10^5^ isolated PBMCs were incubated with 5 µl Human TruStain FcX™ (BioLegend, San Diegeo, USA) for Fc receptor blocking and subsequently stained with anti-CD14-V450 (clone MΦP9), anti-CD16-FITC (clone B73.1), anti CD15-APC-canine 7 (Cy7) (clone W6D3; BioLegend, San Diego, USA), anti-CD11b-Brilliant Violet 605 (clones ICRF44 and M1/70; BioLegend, San Diego, USA), anti-CD33-PerCP-cyanine 5.5 (clone WM53; BioLegend, San Diego, USA), and anti-HLA-DR-APC-R700 (clone G46.6; BD Biosciences, Franklin Lakes, USA). After incubation, cells were washed and resuspended in FACS-Flow prior to acquisition on a FACSLyric™ flow cytometer (BD Biosciences, Franklin Lakes, USA). Analysis was performed using BD FACSuite™ software. After exclusion of doublets, MDSC were identified within the CD16^-^HLA-DR^-^/dim CD11b^+^CD33^+^ population and subdivided into granulocytic MDSC (gMDSC; CD15^+^CD14^-^), monocytic MDSC (mMDSC; CD14^+^CD15^-^), and early-stage MDSC (eMDSC; CD14^-^CD15^-^) (43). Gating strategies for all analyses are shown in **Supplementary Figure 1**.

### 2.7 ChIPmentation

Prior to chromatin immunoprecipitation (ChIP), 7 x 10^6^ PBMCs were crosslinked using formaldehyde at a final concentration of 1% for 8 min at 22 °C. To stop the fixation of proteins linked to the DNA 0.125 M glycine was added to the mix. After washing the cells for three times (500xg, 5 min) and cell lysis (10^6^ cells per 1mL lysis buffer) DNA-shearing was performed using the Bioruptor Pico^®^ (Diagenode, Liège, Belgium) to achieve fragments of 150–200 base pairs. Next, we used an automated system (IP-Star^®^ Compact) for immunoprecipitation, reverse cross-linking, DNA purification, and tagmentation with specific reagent kit (Auto ChIPmentation Kit for Transcription Factors, Diagenode, Liège, Belgium) as well as antibodies against histone 3 (H3 Pan) and trimethylated lysine 4 of histone 3 (both Diagenode, Liège, Belgium), all according to manufacturer`s instructions. The enriched chromatin was stripped prior to end repair and reverse cross link according to the ChIPmentation kit guidelines. Unique dual indexes (UDI) (Diagenode, Liège, Belgium) were used for ChIP DNA library amplification and AMPure XP beads (Beckman Coulter, Brea, USA) for purification. Each library’s quality was analyzed using a High Sensitivity DNA chip for Bioanalyzer (Agilent Technologies. Santa Clara, USA) and Qubit™ assay (Qubit Fluorometric Quantitation, Thermo Fisher Scientific, Waltham, USA) was used for quantification of the amount of obtained ChIP-DNA. ChIP-seq libraries were sequenced on a NextSeq 500 Sequencing System using 150-cycle NextSeq 500/550 v2.5 sequencing reagent kits (Illumina, San Diego, USA).

### 2.8 ChIP-seq reads processing and mapping

After careful quality control with FastQC 0.11.9, ChIP-seq data was trimmed and clipped by Trimmomatic 0.39 (44, 45). Short and low-quality reads were discarded. For reads alignment Bowtie 2 2.3.5.1 mapping software was used with the pre-compiled H. sapiens UCSC hg19 reference genome (http://bowtie-bio.sourceforge.net/bowtie2/index.shtml) (46). Ambiguous and duplicate reads were separated before building ChIP-seq sequence alignment maps. SAMtools 1.9, BEDTools 2.29.2, bedGraphToBigWig 4 and bedToBigBed 2.7 conversion tools were used to generate various file formats required for the following downstream processing steps (47–49).

### 2.9 Blacklisted regions and peak calling

In preparation of the peak calling procedure, anomalous, unstructured, or high signal regions, recommended to be blacklisted by the ENCODE project, were removed sample-wise by utilizing the intersect functionality of BEDTools based on the ENCODE provided hg19 blacklist (https://github.com/Boyle-Lab/Blacklist/tree/master/lists) (50). The signal tracks of H3K4me3 histone modifications were subsequently analyzed by MACS2 2.2.7.1 in comparison to H3 Pan sequencing reads originating from the same sample which were used as control (51). Peak range data were imported to the R/Bioconductor environment (52) along with H3K4me3 and H3 Pan signal tracks.

### 2.10 Differential binding analysis, annotation and functional enrichment

Differential histone modification analyses were performed in R using the Bioconductor packages “DiffBind” 3.10.1, “DESeq2” 1.40.2, “csaw” 1.34.0, “profileplyr” 1.16.0, and “ChIPpeakAnno” 3.34.1 (53, 54). Annotation and enrichment analyses were conducted using the annotation databases “org.Hs.eg.db” 3.17.0, “reactome.db” 1.84.0, and “DO.db” 2.9, together with transcript annotations from “TxDb.Hsapiens.UCSC.hg19.knownGene” 3.2.2. All analyses were performed using the human reference genome assembly GRCh37/hg19. Peak region and signal track data processing was performed in DiffBind. Read counts were normalized using native background normalization (DBA_NORM_NATIVE) with background correction enabled. Differential binding analyses included comparisons between ICU and NST samples within defined conditions, as well as longitudinal comparisons between day 1 (D1) and day 8 (D8) samples from ICU-treated individuals.

A consensus peak set was generated using a minimum overlap threshold of two samples per peak. To investigate signal enrichment patterns, ChIP-seq coverage profiles were generated using dba.plotProfile(). Subsequent downstream analyses were restricted to differentially bound peaks overlapping promoter regions, defined as genomic intervals within ±1 kb of annotated transcription start sites (TSSs). Promoter coordinates were derived from transcript annotations provided by “TxDb.Hsapiens.UCSC.hg19.knownGene”. Peak–promoter overlaps were identified through genomic overlap filtering, and retained peaks were annotated based on TSS-centered information from TSS.human.GRCh37. Differentially bound promoter regions were defined as candidates exhibiting a false discovery rate (FDR; Benjamini–Hochberg adjusted p-value) below 0.05.

Differential peaks overlapping promoter regions were further classified into genomic feature categories (promoters, untranslated regions (UTRs), exons, introns, downstream regions, or intergenic regions) according to a hierarchical annotation scheme. Functional enrichment analyses were then performed for the complete promoter-associated peak set as well as for peaks showing increased or decreased enrichment. Gene Ontology (GO) enrichment analyses for biological process (BP) and cellular component (CC) categories were conducted using getEnrichedGO() with Benjamini–Hochberg multiple-testing correction. Reactome pathway enrichment analyses were performed using getEnrichedPATH() in combination with the Reactome database.

### 2.11 Statistical analysis and data visualization

Statistical analysis and data visualization were performed using GraphPad Prism (Version 6.0f, GraphPad Software, La Jolla, USA). For descriptive statistics, categorial data are presented as number and percent, while continuous data are presented as median and interquartile range (IQR), as indicated. Results for standard laboratory parameters, FACS data and cytokine concentrations are visualized as individual data points, box and whiskers. Between-group comparisons were performed using Fisher’s exact test (reported with odds ratio (OR) and 95% confidence interval (CI)) for categorical data and Mann-Whitney U test for continuous data. Longitudinal changes within the ICU group between T1 and T2 were analyzed using the Wilcoxon signed rank test. P values < 0.05 were considered significant in all analyses and indicated with ‘*’ (p<0.05, >0.01), ‘**’ (p<0.01), ‘***’ (p<0.001) or ‘****’ (p<0.0001), respectively.

## 3 Results

### 3.1 Patients’ characteristics

In total, 46 patients hospitalized due to SARS-CoV-2 infection were enrolled. Based on initial placement after hospital admission, 27 patients (59%) were assigned to the ICU group, and 19 patients (41%) to the non-ICU group. In both groups, blood samples for the experimental workflow were drawn within 24 h after hospitalization (T1). In the ICU group, a second blood sampling was performed after 7 days (T2). Full data sets for epigenetic analyses were available for 17/19 patients in the non-ICU group (T1), for 27/27 patients in the ICU group at T1 and for 14/17 patients in the ICU group at T2 (**Supplementary Figure 2**).

Baseline clinical and sociodemographic characteristics as well as clinical data of all patients are summarized in **Table 1**. No differences were observed between both groups with respect to age and sex. Overall, the patients in the ICU group had a higher body mass index (BMI; 30 (27–34) kg/m^2^ vs. 28 (23–33) kg/m^2^; p = 0.048), Charlson Comorbidity Index (CCI; 1 (1–2) vs. 0 (0–1); p = 0.01), Sequential Organ Failure Assessment (SOFA) score on admission (11 (4–13) vs. 1 (0–1); p < 0.001), as well as longer hospital stay (19 (11–41) days vs. 7 (4–12) days; p < 0.001), and higher in-hospital mortality (33% vs. 0%; OR 20, 95% CI 1.1-371; p = 0.004) compared with patients in the non-ICU-group. Concerning treatment, systemic corticosteroids were administered to all ICU patients (27/27) compared with 8/19 non-ICU patients, while remdesivir (9/27 vs. 0/19), tocilizumab (3/27 vs. 0/19), and vasopressor support at admission (5/27 vs. 0/19) were essentially restricted to the ICU group, in line with the higher level of respiratory support in these patients. Calculated antibiotic therapy during the first week did not differ significantly between groups (9/27 vs. 3/19; p = 0.31).

**Table 1:** Patient characteristics.

Variables	Total (n=46)	ICU (n=27)	Non-ICU (n=19)	OR	CI 95%	p-value
Demographics						
Age, median (IQR)	62 (54–72)	62 (55–77)	62 (52-69)	–	–	0.28
Male sex, n (%)	35 (76)	22 (82)	13 (68)	2.0	0.51–8.0	0.32
BMI, median (IQR)	28 (25-33)	30 (27-34)	28 (23-33)	–	–	0.048
CCI, median (IQR)	1 (0-2)	1 (1-2)	0 (0-1)	–	–	0.01
Max. SOFA, median (IQR)	4 (1-11)	11 (4-13)	1 (0-1)	–	–	<0.001
Treatment						
Systemic corticosteroids, n (%)	35 (76)	27 (100)	8 (42)	inf.	7.8 to inf.	<0.001
Calculated antibiotic therapy (d1–7), n (%)	12 (26)	9 (33)	3 (16)	2.7	0.61-12	0.31
Remdesivir, n (%)	9 (20)	9 (33)	0 (0)	inf.	2.1 to inf.	0.006
Tocilizumab, n (%)	3 (7)	3 (11)	0 (0)	inf.	0.63 to inf.	0.26
Vasopressors on admission, n (%)	5 (11)	5 (19)	0 (0)	inf.	1.1 to inf.	0.07
Respiratory support						
room air/low-flow O_2_, n (%)	20 (43)	1 (4)	19 (100)	0.0	0.0 to 0.02	<0.001
high-flow O_2_/NI, n (%)	22 (48)	22 (81)	0 (0)	inf.	16 to inf.	<0.001
invasive ventilation, n (%)	4 (9)	4 (15)	0 (0)	inf.	0.69 to inf.	0.13
Length of stay and mortality						
LOS, median (IQR)	12 (6-23)	19 (11-41)	7 (4-12)	–	–	<0.001
90-day mortality, n (%)	9 (20)	9 (33)	0 (0)	20	1.1–371	0.004

Data are presented as median (IQR) or n (%). BMI, body mass index; CCI, Charlson comorbidity index; CI, confidence interval; inf., infinity; OR, odds ratio; SOFA, sequential organ failure assessment; LOS, length of stay. Group comparisons were performed using the Mann-Whitney U-test for continuous variables and Fisher’s exact test for categorical variables.

### 3.2 Increased inflammation markers in ICU patients with differential longitudinal changes

**Figure 1:**
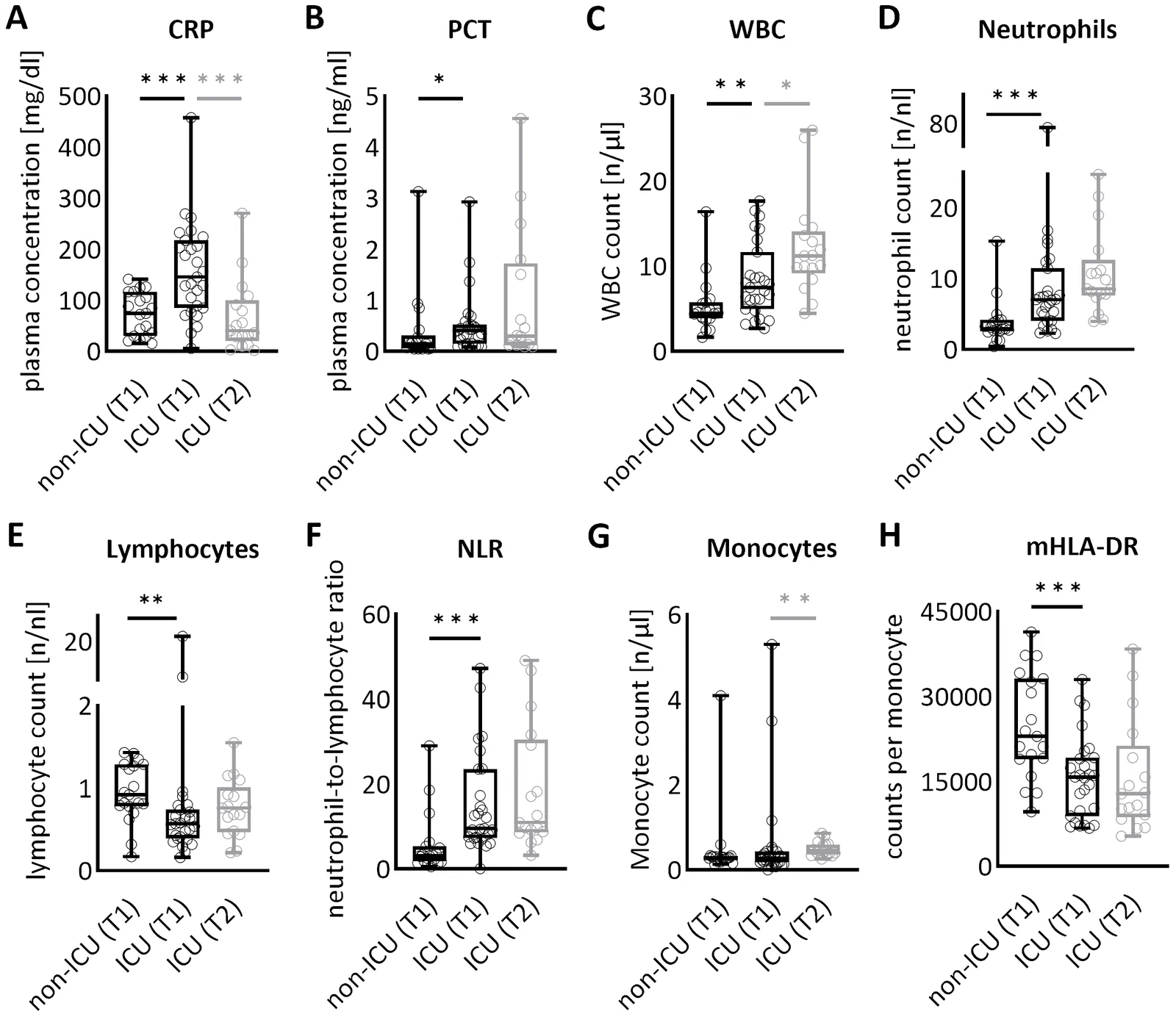
Routine clinical inflammatory biomarkers, differential blood count analysis and monocyte surface HLA-DR expression. **(A)** C-reactive protein (CRP), **(B)** procalcitonin (PCT); **(C)** white blood cell count (WBC), **(D)** neutrophils, **(E)** lymphocytes with **(F)** neutrophil-to-lymphocyte ratio (NLR), **(G)** monocytes and **(H)** density of HLA-DR molecules on monocyte surface were measured on day 1 (T1) in the total cohort and day 7 after study inclusion (T2) in the ICU group. Data are presented as box and whiskers (min to max) with each patient being represented as a single data point. Values were available for n=27 patients in the ICU group at T1, n = 17 patients at T2 and n = 19 patients in the non-ICU group except for PCT (ICU group T2 n = 16 patients; non-ICU group n = 18 patients). Group comparisons were performed by two-sided Mann-Whitney U-test, while longitudinal changes within the ICU group between T1 and T2 were analyzed using the Wilcoxon signed rank test, with `***´ indicating p-values *p* < 0.001 and `**´ indicating p-values < 0.01 and `*´ indicating p-values < 0.05.

When comparing routine clinical inflammatory biomarkers at T1, patients in the ICU group had higher median CRP levels (146 (85–218) vs. 74 (30–117) mg/dl; p = 0.0004), PCT (0.41 (0.15-0.52) vs. 0.15 (0.06-0.31) ng/ml; p = 0.01), and white blood cell counts (7.5 (5.0-12) vs. 4.5 (3.9-5.8) x 10^9^/L; p = 0.006) (**Figures 1A–C**). Differential blood count analysis (**Figures 1D–G**) revealed that the observed leukocytosis in the ICU cohort was predominantly driven by neutrophilia (7.1 (4.0-11) x 10^9^/L vs. 3.0 (2.6-4.2) x 10^9^/L; p = 0.0005), while lymphocyte counts were significantly reduced (0.57 (0.39-0.74) x 10^9^/L vs. 0.92 (0.78-1.3) x 10^9^/L; p = 0.001), resulting in an elevated neutrophil-to-lymphocyte ratio (NLR) (9.5 (7.3-24) x 10^9^/L vs. 3.1 (1.9-5.3); p < 0.0001). Monocyte counts did not differ between groups at T1 (0.27 (0.18-0.43) x 10^9^/L vs. 0.29 (0.25-0.31) x 10^9^/L; p = 0.69).

In the ICU cohort, CRP levels decreased significantly within the observation period (T2: 41 (20–100) mg/dl vs. T1: 146 (85–218) mg/dl; p = 0.005), PCT did not change markedly (T2: 0.30 (0.12-1.7) vs. T1: 0.41 (0.15-0.52) ng/ml; p = 0.40) while the white blood cell count increased (T2: 11 (9.1-14) x10^9^ vs. T1: 7.5 (5.0-12) x 10^9^/L; p = 0.03), with a significant rise in monocyte counts (T2: 0.46 (0.37-0.60) x 10^9^/L vs. T1: 0.27 (0.18-0.43) x 10^9^/L; p = 0.008), while both neutrophil (T2: 8.6 (7.6-13) x 10^9^/L vs. T1: 7.1 (4.0-11) x 10^9^/L; p = 0.22) and lymphocyte counts did not change significantly (T2: 0.76 (0.47-1.0) x 10^9^/L vs. T1: 0.57 (0.39-0.74) x 10^9^/L; p = 0.26). The NLR remained elevated (T2: 11 (8.6-30) vs. T1: 9.5 (7.3-24); p = 0.49), suggesting a transition from an acute-phase systemic inflammatory response to a sustained innate immune response activation characterized by persistent neutrophilia, an emerging monocytosis, and persisting lymphopenia.

### 3.3 Increased granulocytic myeloid-derived suppressor cells in the mononuclear fraction of ICU patients

In critically ill patients with COVID-19, density gradient centrifugation can co-isolate non-mononuclear cells, in particular low-density granulocytes, whose buoyant density overlaps with that of mononuclear cells (55, 56). To characterize the cellular composition of the isolated PBMC fraction and to assess the potential contribution of these co-isolating low-density granulocytes, flow cytometric immunophenotyping was performed, including quantification of monocytes, CD3^+^ T cells and B-cells alongside the MDSC subsets. The gating strategy for the identification of monocyte and lymphocyte subsets is shown in **Supplementary Figure 1**. While the number of monocytes within the isolated PBMC fraction, as determined by flow cytometry, did not differ between non-ICU and ICU groups at T1 (275 (172–462) cells/µl vs. 271 (202–319) cells/µl; p = 0.86), patients in the ICU group showed a markedly increased number of granulocytic myeloid-derived suppressor cells (gMDSC) compared to the non-ICU group (14 (3.7-22) cells/µl vs. 2.1 (0.8-7.0) cells/µl; p = 0.0001). A comparable increase was observed for monocytic MDSC (mMDSC) with a median of 6.7 (1.3-49) cells/µl vs. 0.6 (0.13-4.2) cells/µl (p = 0.01), whereas early-stage MDSC (eMDSC) were lower in the ICU group (3.6 (1.7-9.9) cells/µl vs. 9.6 (5.1-16) cells/µl; p = 0.01). The expansion of gMDSC within the mononuclear fraction parallelled the neutrophilia observed in the whole-blood differential of the same patients, consistent with a disease-associated enrichment of low-density granulocytes that co-purify with mononuclear cells during critical illness. Within the ICU cohort, paired comparison of both time points revealed no significant change in the number of gMDSC within the mononuclear fraction (T2: 11 (2.1-28) cells/µl vs. T1: 14 (3.7-22) cells/µl; p = 0.85), nor did the number of co-isolated monocytes (T2: 354 (213–476) cells/µl vs. T1: 275 (172–462) cells/µl; p = 1.0), indicating a stable cellular composition of the analyzed fraction over the course of the ICU treatment (**Supplementary Figure 3**).

### 3.4 Sustained reduction in monocyte HLA-DR receptor density in ICU patients

Monocyte surface density of HLA-DR molecules was determined via flow cytometry to assess monocyte antigen presentation capacity. At T1, patients in the ICU group showed a reduced HLA-DR receptor density compared to non-ICU patients (15803 (8895–19213) counts per monocyte vs. 22956 (18975–33161) counts per monocyte; p = 0.0007) (**Figure 1H**). ICU patients exhibited persistently reduced monocyte HLA-DR levels at T2 (T2: 12818 (8725–21341) counts per monocyte vs. T1: 15803 (8895–19213) counts per monocyte; p = 0.43), suggesting ongoing immune dysfunction that was not resolved within the first week of critical illness.

### 3.5 Impaired innate immune responsiveness in ICU patients after *ex vivo* stimulation

**Figure 2:**
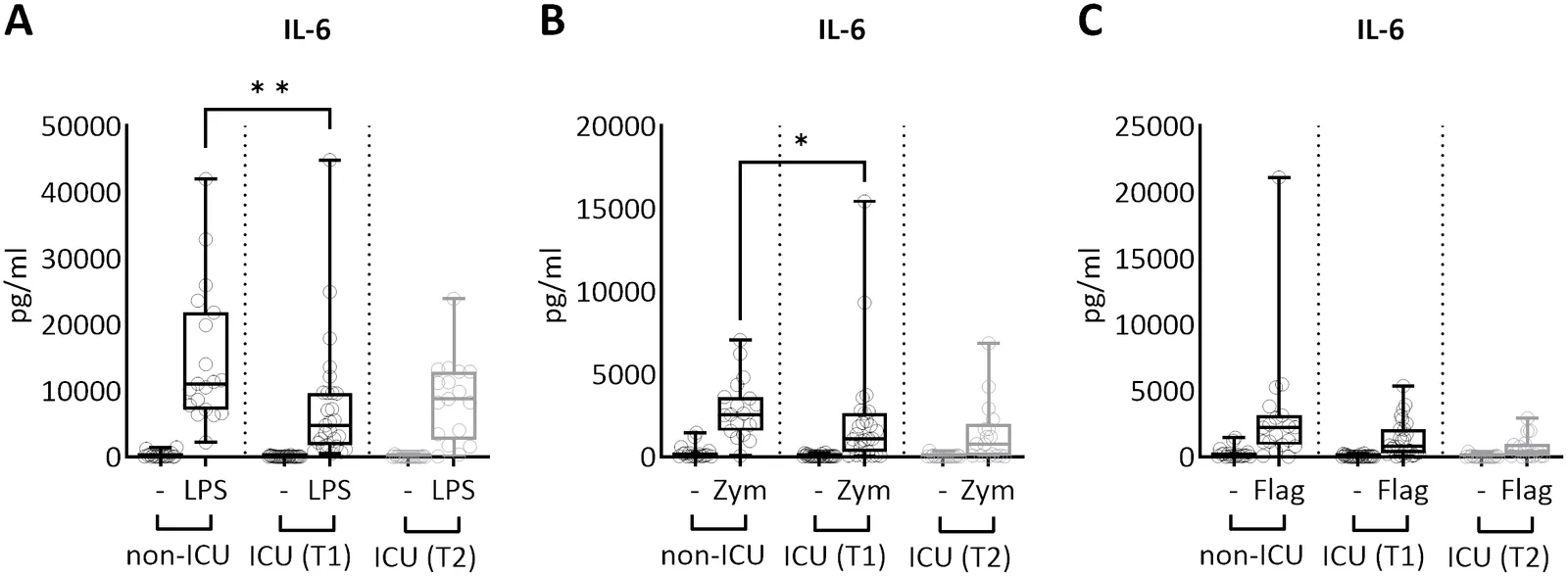
Ex vivo secretion of inflammatory cytokine. IL-6 levels in supernatants from *ex vivo* stimulated and unstimulated samples of ICU and non-ICU patients. IL-6 levels in unstimulated supernatants (`-´) or after ex-vivo stimulation with **(A)** bacterial lipopolysaccharide (LPS), **(B)** Zymosan, and **(C)** Flagellin. Data are presented as box and whiskers (min to max) with each patient being represented as a single data point. Group comparisons between the ICU group (n = 27 patients) and the non-ICU group (n = 19 patients) were performed by two-sided Mann-Whitney U-test. Longitudinal changes in IL-6 secretion within the ICU group between T1 and T2 (n = 17 patients) after ex-vivo stimulation were analyzed using the Wilcoxon signed rank test `**´ indicating p-values p < 0.01 and `*´ p < 0.05.

To analyze functional changes in the innate immune response, *ex vivo* stimulation of PBMCs with Toll-like receptor (TLR) 4 agonist LPS, TLR 5 agonist Flagellin, and TLR 2 agonist Zymosan was performed for 24 h and subsequent IL-6 secretion was measured. Compared to the non-ICU group, patients in the ICU group exhibited a reduced secretion of IL-6 after stimulation with LPS (4750 (1785–9683) pg/ml vs. 11054 (7113–21803) pg/ml; p = 0.002) and Zymosan (1085 (311–2635) pg/ml vs. 2551 (1565–3622) pg/ml; p = 0.02) stimulation, whereas no between-group differences were observed after stimulation with Flagellin at T1 (**Figures 2A–C**). Within the ICU group, ex vivo IL-6 secretion did not change significantly between T1 and T2 for any of the three stimuli (LPS, p = 0.93; Zymosan, p = 0.46; Flagellin, p = 0.10; Wilcoxon signed-rank test), indicating that the impaired immune responsiveness present at admission persisted throughout the first week of critical illness.

### 3.6 Genome wide differential binding of H3K4me3 between ICU and non-ICU patients

**Figure 3:**
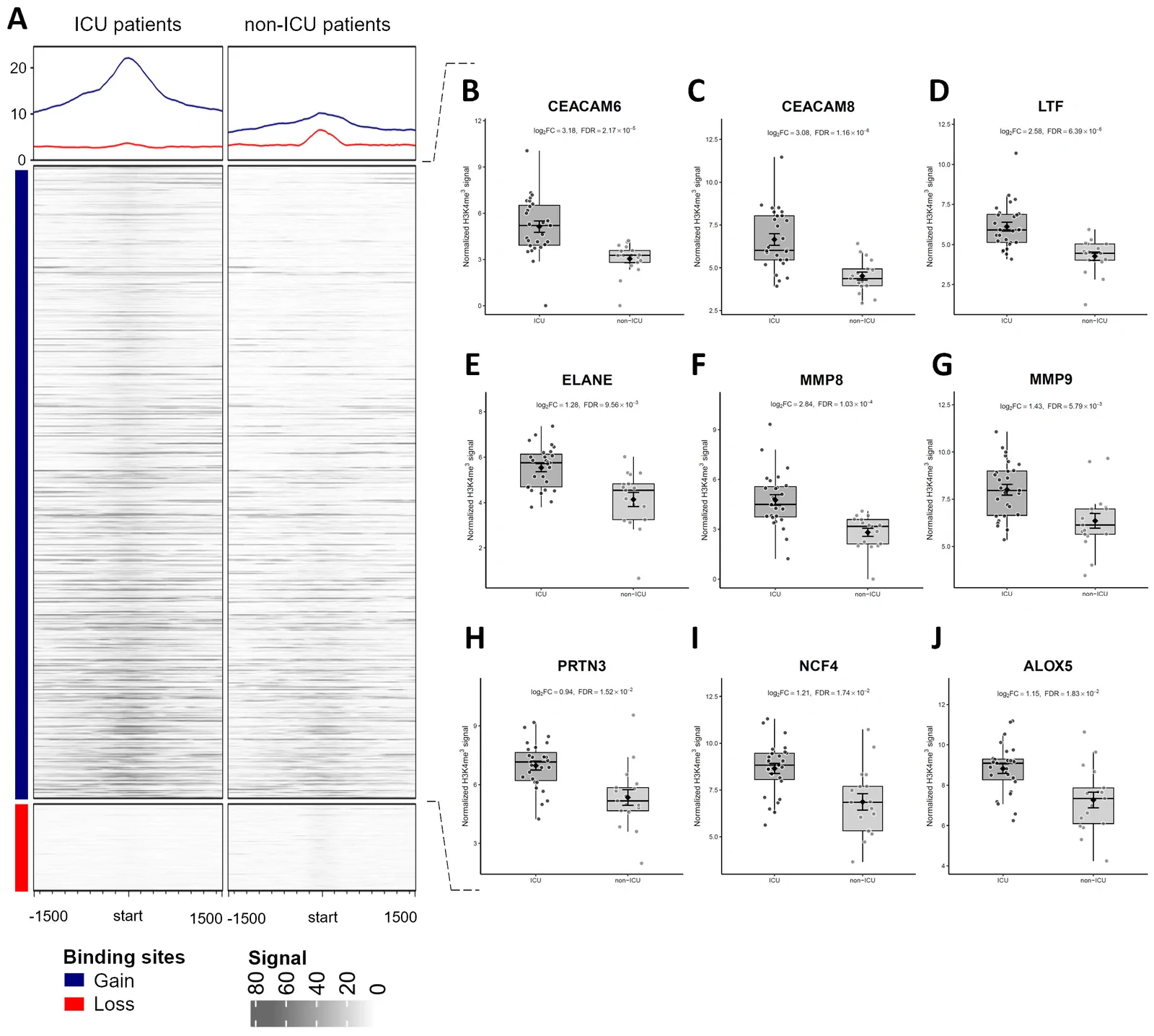
Differential H3K4me3 enrichment in ICU patients and non-ICU patients at hospitalization (T1). **(A)** Heatmaps and normalized histone modification profiles show H3K4me3 enrichment at regions identified as differentially bound between the two groups by DiffBind analysis. Graphs above the heatmaps summarize the average H3K4me3 enrichment across all differentially bound regions. Individual rows in the heatmap represent differentially bound consensus peaks showing either a gain (indicated in dark blue) or loss (indicated in red) of H3K4me3 enrichment. Differences in signal intensity indicate relative increases or decreases in H3K4me3 enrichment at individual genomic regions. **(B–J)** Among the 3714 differentially bound consensus peak regions, H3K4me3 analysis identified 706 promoter-associated peaks (FDR < 0.05), of which 704 showed a higher H3K4me3 binding in the ICU cohort compared to the non-ICU cohort at T1. Increased H3K4me3 enrichment in promoter regions of genes related with neutrophil activation (i.e. *CEACAM6*, *CEACAM8*, *LTF*; **(B–D)**, neutrophil extracellular traps (i.e. *ELANE*, *MMP8*, *MMP9*, *PRTN3*; E-H) as well as oxidative stress (*NCF4*, *ALOX5*; **(I, J)** was observed in the ICU patients compared to the non-ICU patients. Individual points represent samples. Boxes indicate the median and interquartile range (IQR); whiskers extend to the most extreme data points within 1.5 × IQR. Black diamonds indicate the group mean, and error bars represent the standard error of the mean (SEM).

Genome-wide H3K4me3 ChIP-seq analysis revealed a differential binding pattern between the ICU group and non-ICU group at hospital admission (T1; **Figure 3**; **Supplementary Figures 4**, **5**). In total, 3714 regions displayed consensual differential H3K4me3 enrichment, including 3577 regions with increased enrichment and 137 regions with reduced enrichment in the ICU cohort (**Supplementary Figure 6A**). Genomic feature annotation of promoter-associated differential peaks showed that most peak regions were classified as promoter regions (**Supplementary Figure 6B**).

### 3.7 Increased H3K4me3 occupancy in critically ill individuals at T1 is consistent with neutrophil hyperactivation

Among the 3714 differentially bound consensus peak regions, H3K4me3 analysis identified 706 promoter-associated peaks (FDR < 0.05), of which 704 showed a higher H3K4me3 binding in the ICU cohort compared to the non-ICU cohort at T1. Increased H3K4me3 enrichment in promoter regions of genes related with neutrophil activation (i.e. *CEACAM6*, *CEACAM8*, *LTF*; **Figures 3B–D**), neutrophil extracellular traps (i.e. *ELANE, MMP8, MMP9, PRTN3*; **Figures 3E–H**) as well as oxidative stress (*NCF4, ALOX5*; **Figures 3I, J**) was observed in the ICU patients compared to the non-ICU patients. Of note, only a single gene region, linked to immune-related function, showed increased H3K4me3 enrichment in the non-ICU group (*CHI3L2*). Notably, neither the IL6 gene itself nor genes related to IL-6 signaling (including *IL6R*, *IL6ST*, *JAK1*, *JAK2*, *STAT1*, *STAT3*, *SOCS1*, *SOCS3*, NF-κB subunits, *TLR4*, *MYD88*, and *NLRP3*) showed differential H3K4me3 enrichment at their promoter regions between ICU and non-ICU patients at T1.

**Figure 4:**
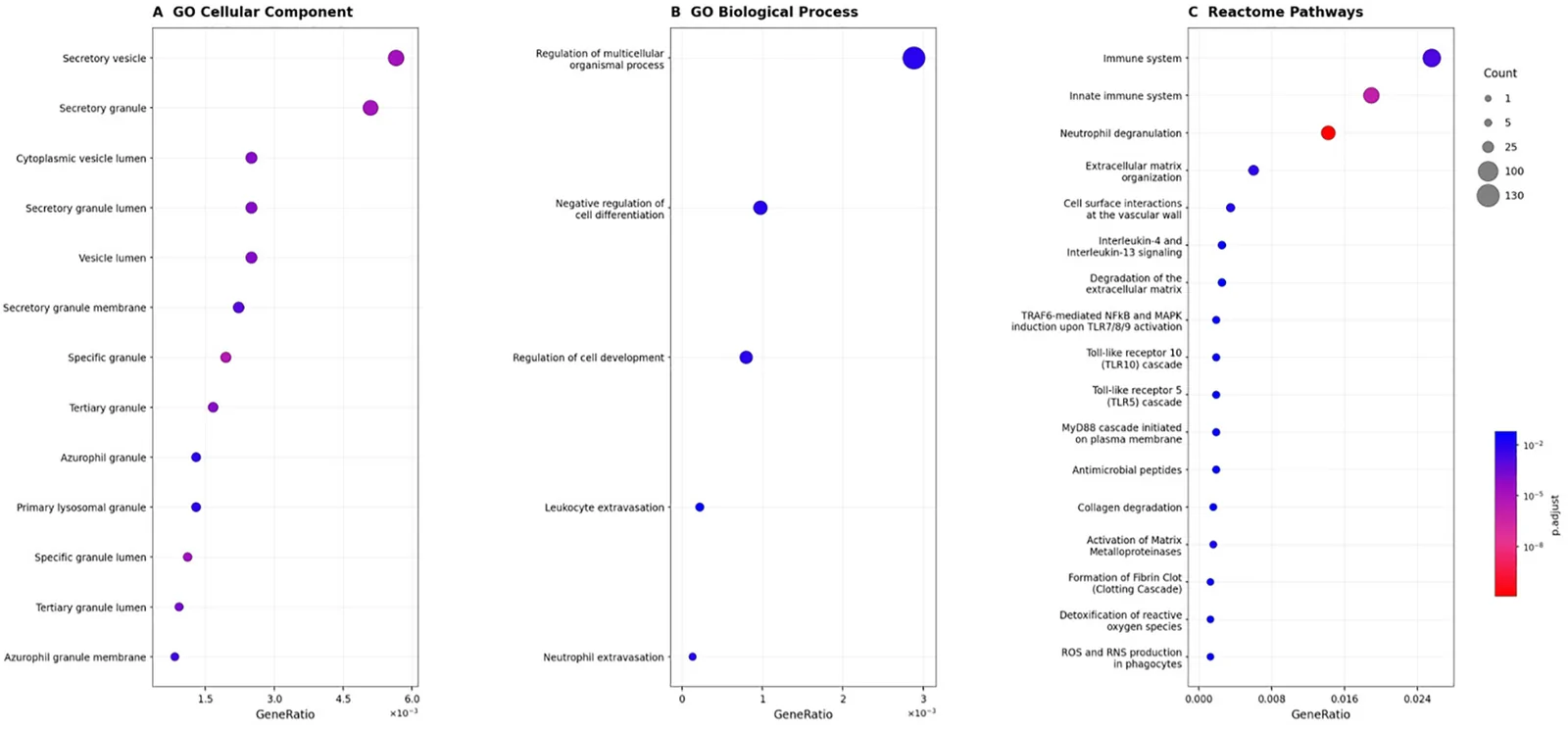
Gene Ontology and Reactome pathway enrichment analysis of differentially bound H3K4me3 peaks between ICU and non-ICU patients at T1. Enrichment analysis of genes associated with increased H3K4me3 occupancy in ICU patients (706 differentially bound peaks, FDR < 0.05, mapping to 568 unique genes). (A+B) Multi-panel dot plot showing enrichment results across three databases. **(A)** Gene Ontology Cellular Component (GO CC). **(B)** Gene Ontology Biological Process (GO BP). **(C)** Filtered Reactome dot plot displaying all immunologically relevant pathways, sorted by gene ratio. Across all panels, the x-axis represents the gene ratio (number of genes in the dataset annotated to a given pathway divided by the total number of annotated genes), dot size reflects the gene count per pathway, and dot color indicates statistical significance (p.adjust; red = highly significant, blue = less significant).

Subsequent GO-term analysis of genes associated with increased H3K4me3 occupancy in ICU patients revealed several immune-related cellular components (CC). All CC terms were associated with neutrophil granulation (**Figure 4A**; **Supplementary Table 1**). Among biological processes both leucocyte and neutrophil extravasation (GO:0045123; GO:0072672) and several cell regulatory and developmental progresses were significantly enriched (**Figure 4B**; **Supplementary Table 2**). Additionally, KEGG pathway analysis showed significant enrichment of inflammatory and innate immune response pathways among gene regions with increased H3K4me3 binding in ICU patients. Significant pathways associated with immune function were neutrophil degranulation (R-HSA-6798695), followed by innate immune system (R-HSA-168249), immune system (R-HSA-168256) and ROS and RNS production in phagocytes (R-HSA-1222556) (**Figure 4C**).

### 3.8 Longitudinal changes in genome wide binding of H3K4me3 in ICU patients

**Figure 5:**
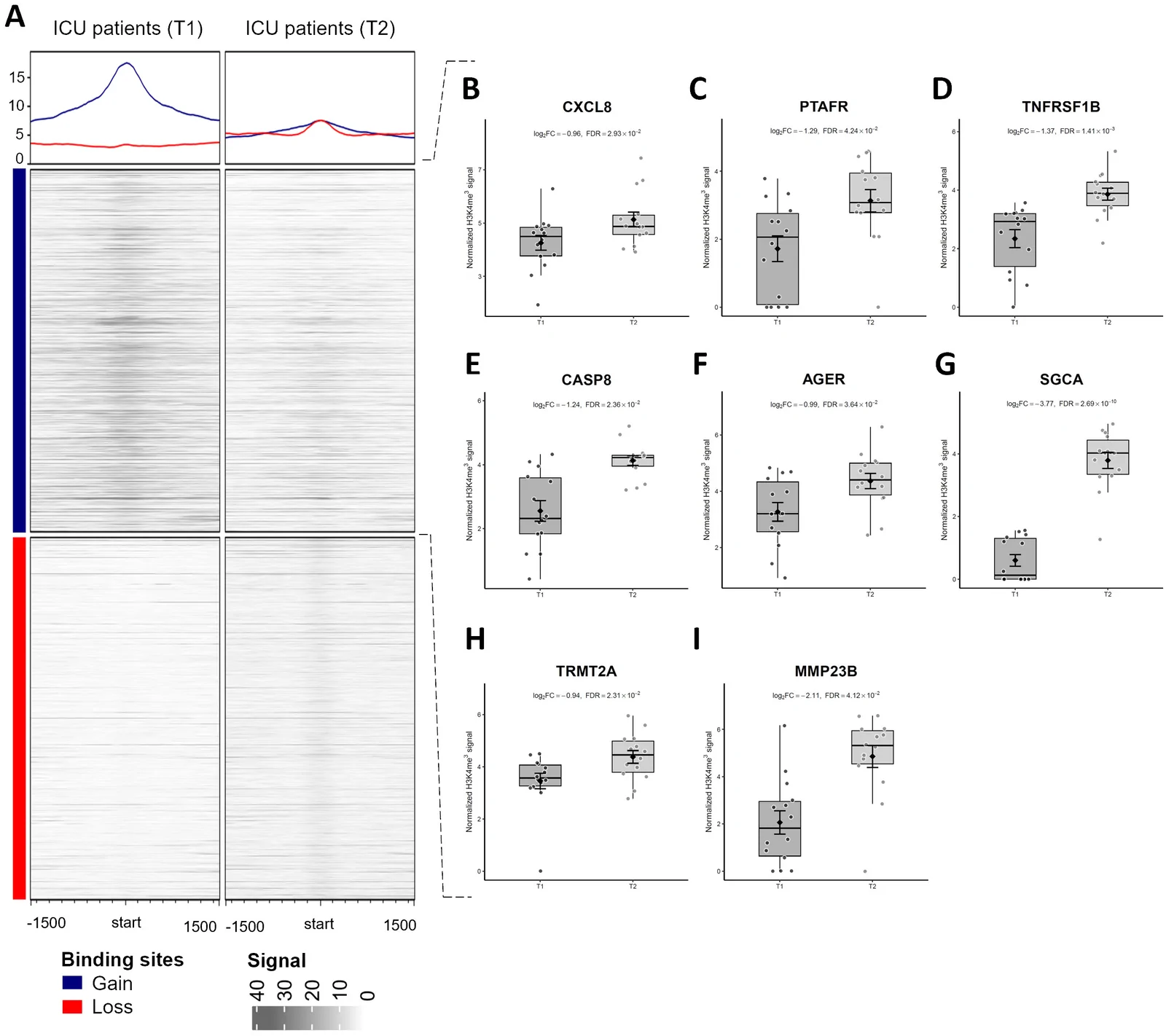
Differential H3K4me3 enrichment in ICU patients at T1 and T2. **(A)** Heatmaps and normalized histone modification profiles show H3K4me3 enrichment at genomic regions identified as differentially bound between the two time points by DiffBind analysis. Graphs above the heatmaps summarize the average H3K4me3 enrichment or loss of enrichment across all differentially bound regions. Individual rows in the heatmap represent differentially bound consensus peaks showing either a gain or loss of H3K4me3 enrichment. Differences in signal intensity indicate relative increases or decreases in H3K4me3 enrichment at individual genomic regions. **(B–I)** Analysis of H3K4me3 enrichment at T2 revealed 270 peaks with increased occupancy compared to T1, including neutrophil-associated loci [i.e., *CXCL8*, *PTAFR*; **(B, C)**] and inflammatory mediators [i.e. *TNFRSF1B*, *CASP8*, *AGER*; **(D–F)**], consistent with a transition from antiviral immune response to a sustained innate immune activation. Additionally, promoter regions of genes responsible for tissue remodeling showed an increased enrichment in the later phase of the disease process [i.e. *SGCA*, *TRMT2A*, *MMP-23B*; **(G–I)**]. Individual points represent samples. Boxes indicate the median and interquartile range (IQR); whiskers extend to the most extreme data points within 1.5 × IQR. Black diamonds indicate the group mean, and error bars represent the standard error of the mean (SEM).

When comparing ICU patients between T1 and T2, a total of 8950 differentially enriched consensus regions were detected. Among these, 6311 regions showed a significant increase and 2639 regions showed a significant reduction in H3K4me3 enrichment (**Figure 5**; **Supplementary Figures 7**, **8**). As in the baseline comparison, genomic feature annotation of the promoter-associated differential peaks revealed a predominant classification as promoter regions (**Supplementary Figure 7B**).

### 3.9 Temporal changes in H3K4me3 binding profile reveal shift from antiviral interferon response to sustained innate immune response and tissue remodeling

**Figure 6:**
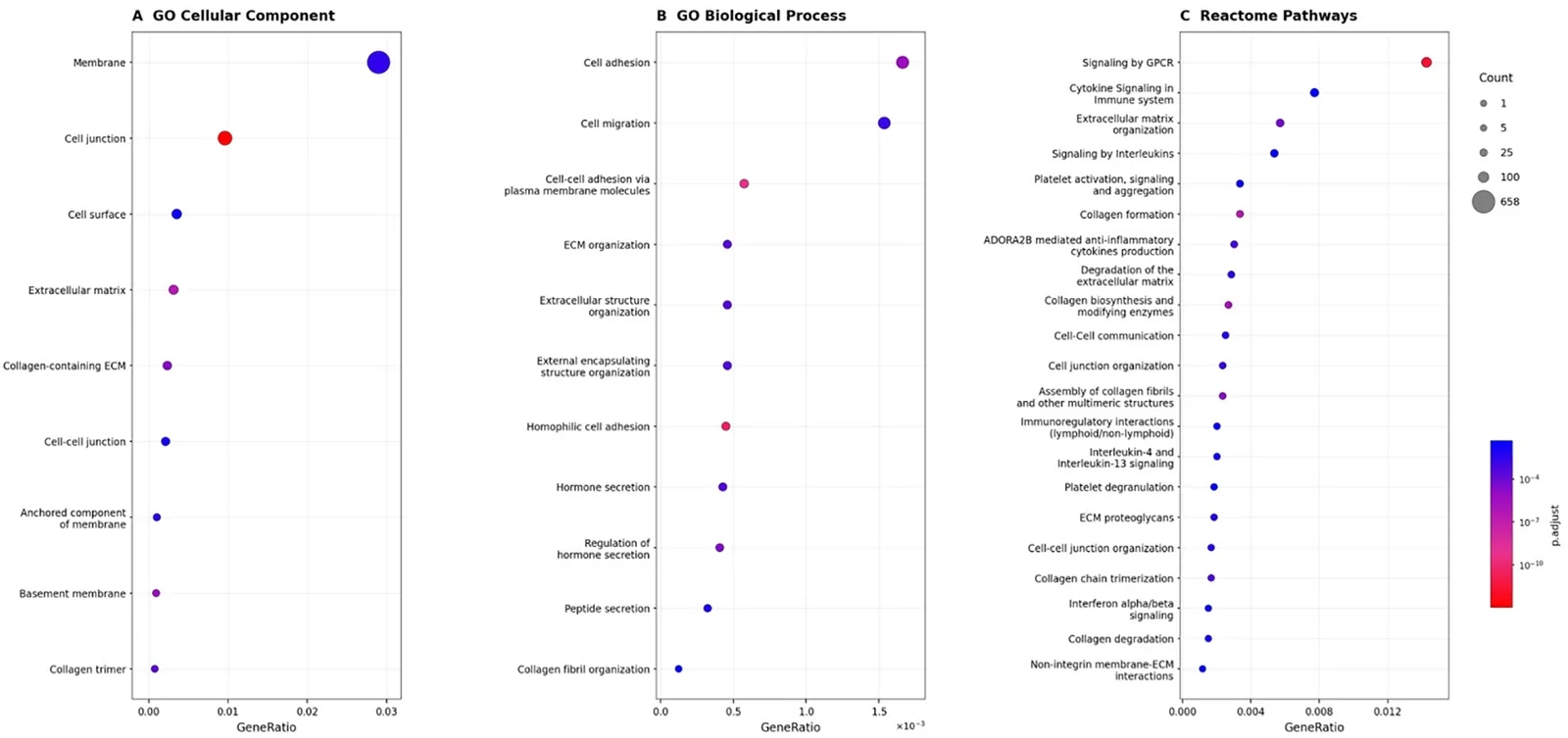
Gene Ontology and Reactome pathway enrichment analysis of differentially bound H3K4me3 peaks in ICU patients between T1 and T2. Enrichment analysis of genes associated with differentially bound H3K4me3 peaks between ICU admission (Day 1) and Day 8 (1865 peaks, FDR < 0.05). (A+B) Multi-panel dot plot showing enrichment results across three databases. **(A)** Gene Ontology Cellular Component (GO CC) **(B)** Gene Ontology Biological Process (GO BP) **(C)** Filtered Reactome dot plot displaying all immunologically relevant pathways sorted by gene ratio. Across all panels, the x-axis represents the gene ratio, dot size reflects the gene count per pathway, and dot color indicates statistical significance (p.adjust; red = highly significant, blue = less significant).

In the ICU cohort, H3K4me3 binding profile analysis comparing T2 with T1 revealed 1865 differently bound consensual peaks associated to promoters (FDR < 0.05), with 1595 showing higher H3K4me3 enrichment at T1. H3K4me3 profiling at T1 revealed a broad antiviral immune response at the onset of critical illness by increased H3K4me3 enrichment at promoter regions of interferon-stimulated genes (i.e. *IFI44L*, *IFI27*, *IFIT1*, *CMPK2*), members of the 2’-5’-oligoadenylate synthetase family (*OAS1, OAS2, OAS3*), as well as the master regulator of type I interferon production (*IRF7*). Analysis of H3K4me3 enrichment at T2 revealed 270 peaks with increased occupancy compared to T1, including neutrophil-associated loci (i.e., *CXCL8*, *PTAFR*; **Figures 5B, C**) and inflammatory mediators (i.e. *TNFRSF1B*, *CASP8*, *AGER*; **Figures 5D–F**), consistent with a transition from antiviral immune response to a sustained innate immune activation. Additionally, promoter regions of genes responsible for tissue remodeling showed an increased enrichment in the later phase of the disease process (i.e. *SGCA*, *TRMT2A*, *MMP-23B*; **Figures 5G–I**). Although no immune-specific GO terms reached significance, CC analysis identified extracellular matrix (ECM)-associated terms enriched, including extracellular matrix (GO:0031012), basement membrane (GO:0005604), and collagen-containing ECM (GO:0062023) (**Figure 6A**). Furthermore, analysis of biological processes indicated significant enrichment for ECM organization (GO:0030198) and lung development (GO:0030324), suggesting activation of regenerative process in critically ill patients already at admission (**Figure 6B**). KEGG-pathway analysis supported this pattern, with GPCR signaling (R-HSA-372790) and collagen formation (R-HSA-1474290) being the two of the most enriched pathways, indicating epigenetic priming for structural remodeling processes at ICU admission. Immune related pathways with increased enrichment like interferon alpha/beta signaling (R-HSA-909733) were consistent with a counter regulatory immune response to the initial hyperinflammatory state (**Figure 6C**).

## 4 Discussion

Changes in histone modification patterns at gene promoters have been identified as a key mechanism of innate immune cell dysfunction in critical illness, directly linking epigenetic reprogramming to impaired immune responsiveness (31, 36). In the present study, we describe the genome-wide H3K4me3 landscape of circulating innate immune cells in patients hospitalized due to COVID-19 and demonstrate that disease severity is associated with distinct chromatin changes that reflect both the degree and longitudinal dynamics of innate immune dysregulation. The observed H3K4me3 alterations showed differential enrichment at gene loci associated with neutrophil activation and degranulation, NET formation, and oxidative burst in ICU patients compared to patients with moderate disease course, while longitudinal profiling revealed a shift from an early antiviral interferon-driven chromatin state at ICU admission towards sustained innate immune activation and tissue remodeling by day seven. These findings are in line with transcriptomic studies demonstrating that severe COVID-19 is characterized by dysregulation of circulating monocytes and neutrophils, including dysfunctional HLA-DR-low monocytes and neutrophil precursors indicative of emergency myelopoiesis, both of which were absent in patients with mild disease (57). Providing a direct chromatin-level correlate of this predicted epigenetic reprogramming through genome-wide H3K4me3 profiling, our findings also complement recent transcriptomic work that identified a specific metabolic and epigenetic imprint in circulating immune cells of critical COVID-19 patients (39). Furthermore, these findings extend prior epigenomic work showing extensive chromatin remodeling in monocytes from patients with acute severe COVID-19, with differentially accessible regions correlating with transcriptional changes in immune regulatory genes (58, 59). Taken together, our data provides the first genome-wide H3K4me3 map of circulating immune cells during acute critical COVID-19 illness and suggests that epigenetic reprogramming is not only a post-infectious phenomenon, but an active process already detectable within the first days of critical illness.

Among the differentially bound H3K4me3 regions, 704 of 706 peaks showed increased enrichment in the ICU group, mapping predominantly to genes associated with neutrophil degranulation, NET formation, and oxidative burst. Increased H3K4me3 enrichment at promoters of *CEACAM6* and *CEACAM8*, which are established neutrophil activation antigens stored in specific and azurophilic granules and upregulated upon stimulation, is consistent with an epigenetically primed state for granule release and neutrophil-endothelial adhesion in critically ill patients (60). Similarly, increased H3K4me3 occupancy at the promoters of *ELANE*, *PRTN3*, *MMP8*, and *MMP9* reflects epigenetic priming for the release of proteolytic enzymes implicated in NET formation, extracellular matrix degradation, and alveolar barrier disruption in severe COVID-19, consistent with elevated plasma levels of these proteins reported in critically ill patients (21–23, 61). Elevated H3K4me3 at the *NCF4* promoter, essential for ROS generation, and *ALOX5*, an enzyme involved in leukotriene synthesis and oxidative stress responses, further supports epigenetic upregulation of oxidative burst pathways in ICU patients, in line with reports of markedly elevated neutrophil-derived ROS production in severe COVID-19 cases (62, 63). In accordance with these gene-level findings, GO-term analysis of cellular components revealed exclusive enrichment of neutrophil granulation-associated terms among the differentially bound regions, while biological process analysis identified significant enrichment of leukocyte and neutrophil extravasation. KEGG pathway analysis supported this pattern, with neutrophil degranulation, innate immune system, and ROS and RNS production in phagocytes among the most significantly enriched pathways in the ICU group. These findings are consistent with transcriptomic and proteomic studies in severe COVID-19, in which neutrophil degranulation consistently emerges as one of the most enriched pathways when comparing severe to mild or non-ICU disease courses (64, 65). Additionally, longitudinal whole blood transcriptome analysis has identified a neutrophil-associated gene module including *LTF*, *ELANE*, *CEACAM8*, and *MMP8* that correlated strongly with WHO disease severity and leukocyte counts over the disease course in severe COVID-19 patients (66) – a transcriptional pattern similar to the epigenetic priming at the same gene loci observed in our data. Taken together, the consistent evidence from individual gene loci, GO-term enrichment, and pathway analysis indicates that increased H3K4me3 enrichment in critically ill COVID-19 patients reflects a broad epigenetic priming of neutrophil effector programs, which is in accordance at the clinical level with rising leukocyte counts and sustained inflammatory markers observed in our cohort over the first week of ICU treatment compared to initial presentation in moderate COVID-19 disease cases. This predominance of chromatin opening at innate immune effector loci is more consistent with epigenetic priming than with the selective gene repression of classical endotoxin tolerance (36). It differs, however, from trained immunity in the strict sense, which requires a return to baseline between stimuli and enhanced responsiveness upon restimulation, whereas our ICU patients showed reduced monocyte HLA-DR expression and diminished *ex vivo* cytokine secretion, suggesting a coexistence of neutrophil effector priming and functional monocyte immunosuppression (38).

Given the pronounced enrichment of H3K4me3 at neutrophil effector gene loci in ICU patients, the contribution of co-isolating granulocytes to the analyzed fraction has to be considered. Density gradient centrifugation of blood from critically ill patients is known to co-isolate low-density granulocytes, whose buoyant density resembles that of mononuclear cells, with this contamination being more pronounced in severe inflammatory states such as sepsis and COVID-19 (55, 56). Consistent with this, patients in the ICU group showed an increased number of gMDSC within the mononuclear fraction, paralleling the neutrophilia observed in the whole-blood differential. The increased H3K4me3 enrichment at neutrophil effector gene loci therefore likely reflects, at least in part, the epigenetic state of disease-associated low-density neutrophils that co-purify with mononuclear cells during critical illness. Rather than representing a mere technical artifact, the expansion of low-density granulocytes within the mononuclear compartment is itself a recognized feature of severe COVID-19 and correlates with disease severity (56, 57, 67); suggesting that their chromatin signature carries biologically relevant information on the dysregulated innate immune response in critical illness. While the gMDSC subset, representing the disease-relevant low-density granulocyte population, accounted for a median of approximately 1.6% of mononuclear cells in the ICU group, this frequency does not reflect the total granulocyte content of the analyzed fraction, as no CD15^+^ depletion or cell-type-specific sorting was performed prior to chromatin immunoprecipitation, and the total low-density granulocyte content was therefore not separately quantified. At the same time, flow cytometric immunophenotyping confirmed that the isolated fraction contained substantial bona fide mononuclear populations, including monocytes, CD3^+^ T cells, and B cells, indicating that it was not dominated by granulocytes alone. Furthermore, the cellular composition of the mononuclear fraction did not change significantly between T1 and T2, indicating that the longitudinal shift in the H3K4me3 landscape from an interferon-driven signature towards sustained innate immune activation cannot be attributed to a time-dependent change in low-density granulocyte contamination. Taken together, while the use of bulk PBMCs without prior cell-type-specific sorting precludes a definitive attribution of the observed chromatin signals to a single leukocyte subset, the co-isolated low-density granulocytes represent a disease-relevant cell population, and depletion of CD15^+^ granulocytes prior to chromatin immunoprecipitation, as previously described (55), would be a valuable refinement for future studies.

The reduced monocyte surface density of HLA-DR molecules in our cohort of ICU patients, compared to patients on the normal ward, which persisted without recovery over the first week of critical illness, is consistent with the functional immunosuppression seen in viral sepsis and supports the concept of sustained monocyte dysfunction in severe COVID-19 (27–30, 68). The concurrent reduction in *ex vivo* IL-6 secretion after LPS and Zymosan stimulation in ICU patients at admission indicates a receptor-specific dysfunction of innate immune responsiveness. This pattern is consistent with the endotoxin-tolerance model in which repeated pathogen-associated stimulation leads to selective epigenetic silencing of pro-inflammatory genes while other pathways are preserved (36). The absence of differential H3K4me3 enrichment at the IL6 locus and its signaling axis indicates that the reduced cytokine responsiveness in ICU patients is not accompanied by differential H3K4me3 enrichment at these genes, supporting the interpretation of the epigenetic and functional alterations seen in the present study as parallel rather than causally linked readouts of immune dysregulation. The relatively small number of peaks with reduced H3K4me3 enrichment in ICU patients – 137 compared to 704 with increased enrichment – stands in contrast to the pronounced functional immunosuppression observed in this cohort. However, in endotoxin tolerance models, it has been demonstrated that loss of H3K4me3 binding at a limited number of functionally critical loci is sufficient to substantially impair immune responsiveness (36). Potential dilution of monocyte-specific epigenetic signals by co-isolated lymphocytes may have limited the detection of H3K4me3 reductions at immunologically relevant loci such as the MHC II region in our cohort, as has previously been shown in septic patients (31). Taken together, the functional data presented is consistent with a state of impaired monocyte antigen presentation capacity in critically ill COVID-19 patients that is established at ICU admission and is not resolved within the first week of treatment.

Longitudinal H3K4me3 profiling between day one and day seven in ICU patients revealed a pronounced shift in chromatin landscape, with 1595 peaks showing higher H3K4me3 binding at T1 compared to 270 peaks at T2. The chromatin signature at ICU admission was characterized by increased H3K4me3 occupancy at promoters of interferon-stimulated genes, including *IFI44L*, *IFI27*, *IFIT1*, *OAS1*, *OAS2*, *OAS3*, *CMPK2*, and the master regulator of type I interferon production *IRF7*, consistent with an active antiviral innate immune response at the onset of critical illness. Type I interferon signaling, mediated through JAK-STAT pathway and induction of OAS family members and IRF7-regulated genes, represents a central early defense mechanism against SARS-CoV-2 replication, and its epigenetic priming at admission is consistent with the active antiviral replication typically present in the early phase of critical COVID-19 (69). Pathway analysis further identified interferon alpha/beta signaling among the most significantly enriched KEGG pathways at day one, consistent with transcriptomic studies in hospitalized COVID-19 patients with similar interferon-associated gene expression in the early phase of disease before diminishing over time (66). By T2, this interferon-driven chromatin signature had markedly contracted, while H3K4me3 enrichment at loci associated with neutrophil recruitment and sustained innate activation had increased, including *CXCL8* and *PTAFR*, and the inflammatory mediators *TNFRSF1B* and *CASP8*. *CXCL8* has been implicated to drive an autocrine feedback loop that reinforces neutrophil-driven immunopathology and NET formation in severe COVID-19 (70). Furthermore, GO-term and KEGG-pathway analysis at T2 showed H3K4me3 enrichment of immune-related pathways including innate immune activation, in line with the rising leukocyte counts observed between T1 and T2 in our cohort. Taken together, the temporal shift in H3K4me3 landscape mirrors the clinical disease course observed in our patients, suggesting a transition from an acute antiviral interferon response towards a sustained innate immune activation over the course of ICU treatment.

In addition to the immunological shifts described above, H3K4me3 enrichment at T2 also included increased occupancy at promoters of genes involved in extracellular matrix remodeling and structural lung repair, including *SGCA*, *TRMT2A*, and *MMP23B*. Furthermore, GO-term analysis identified significant enrichment of ECM-associated cellular components including extracellular matrix, basement membrane, and collagen-containing ECM, while biological process analysis showed enrichment for ECM organization and lung development. KEGG pathway analysis further supported this pattern, with GPCR signaling and collagen formation among the most enriched pathways at T2. ECM remodeling is a known feature of ARDS-associated lung injury, where active collagen synthesis has been detected as early as the exudative phase and increases progressively over the course of mechanical ventilation (71, 72). In severe COVID-19, increased deposition of collagen, fibronectin, and TGF-ß in lung tissue has been reported to exceed that observed in non-COVID-19 ARDS, with a progression from fibrillogenesis-related to collagen production-related gene expression over the course of mechanical ventilation (73). The epigenetic priming of ECM-associated genes in circulating immune cells observed on T2 in our cohort suggests that chromatin remodeling in peripheral blood cells may reflect or contribute to the onset of structural repair processes in the injured lung. Whether this epigenetic signature in PBMCs represents a systemic correlation of lung remodeling or an independent process requires further investigation.

The present study has several limitations. As a prospective single-center observational study with a limited sample size, generalizability may be limited. Treatment was not randomized, and treatment intensity paralleled disease severity. Concurrent therapies, in particular systemic corticosteroids, which were administered more frequently in the ICU group and can reduce monocyte HLA-DR expression, may have contributed to the observed differences and cannot be fully separated from the effects of disease severity itself. The observed differences should therefore be interpreted as associations rather than as effects of SARS-CoV-2 infection alone. Moreover, ChIP-seq was performed on bulk PBMCs rather than isolated immune cell populations. As discussed above, this precludes a definitive attribution of the observed chromatin signals to a single leukocyte subset, particularly in light of the co-isolation of low-density granulocytes in critically ill patients; depletion of CD15^+^ cells or cell-type-specific sorting prior to chromatin immunoprecipitation would be a valuable refinement for future studies. In addition, H3K4me3 was profiled as a single activating histone mark; the integration of complementary epigenetic layers, including DNA methylation and genome-wide chromatin accessibility (e.g., ATAC-seq), together with paired transcriptomic data, would provide a more comprehensive picture of the epigenetic reprogramming in critical COVID-19 and represents an important direction for future work. Finally, longitudinal profiling was limited to ICU patients, and the study cohort was recruited exclusively during the pre-vaccination phase of the pandemic, which may limit the applicability of these findings to vaccinated individuals or infections with later SARS-CoV-2 variants.

In summary, this study provides the first genome-wide characterization of H3K4me3 in circulating immune cells during acute critical COVID-19 illness. The epigenetic landscape of ICU patients was dominated by broad chromatin opening at neutrophil effector gene loci, reflecting the neutrophil hyperactivation that characterized the clinical course of these patients. Longitudinal profiling revealed a transition from an interferon-driven antiviral chromatin signature at ICU admission toward sustained innate immune activation by day seven. Taken together these findings demonstrate that epigenetic reprogramming of circulating immune cells is an active and dynamic process during critical COVID-19, and suggests that H3K4me3 profiling of peripheral blood cells may serve as a tool to characterize the immune state of critically ill patients at the chromatin level. Further studies integrating cell type-specific epigenomic approaches with paired transcriptomic data will be needed to further dissect the mechanistic relationship between chromatin remodeling and functional immune dysregulation in viral sepsis.

## Data Availability

The datasets presented in this study can be found in online repositories. The names of the repository/repositories and accession number(s) can be found below: GSE339365 (GEO).
