# Supplementary material for: Genome-wide H3K4me3 profiling of circulating immune cells reveals dynamic epigenetic reprogramming during acute critical COVID-19
Source: Front Immunol. 2026 Aug 20;17:1907275. doi: 10.3389/fimmu.2026.1907275 (PMC13538114; doi:10.3389/fimmu.2026.1907275)
Supplement: Supplementary file 1 [file DataSheet1.pdf]

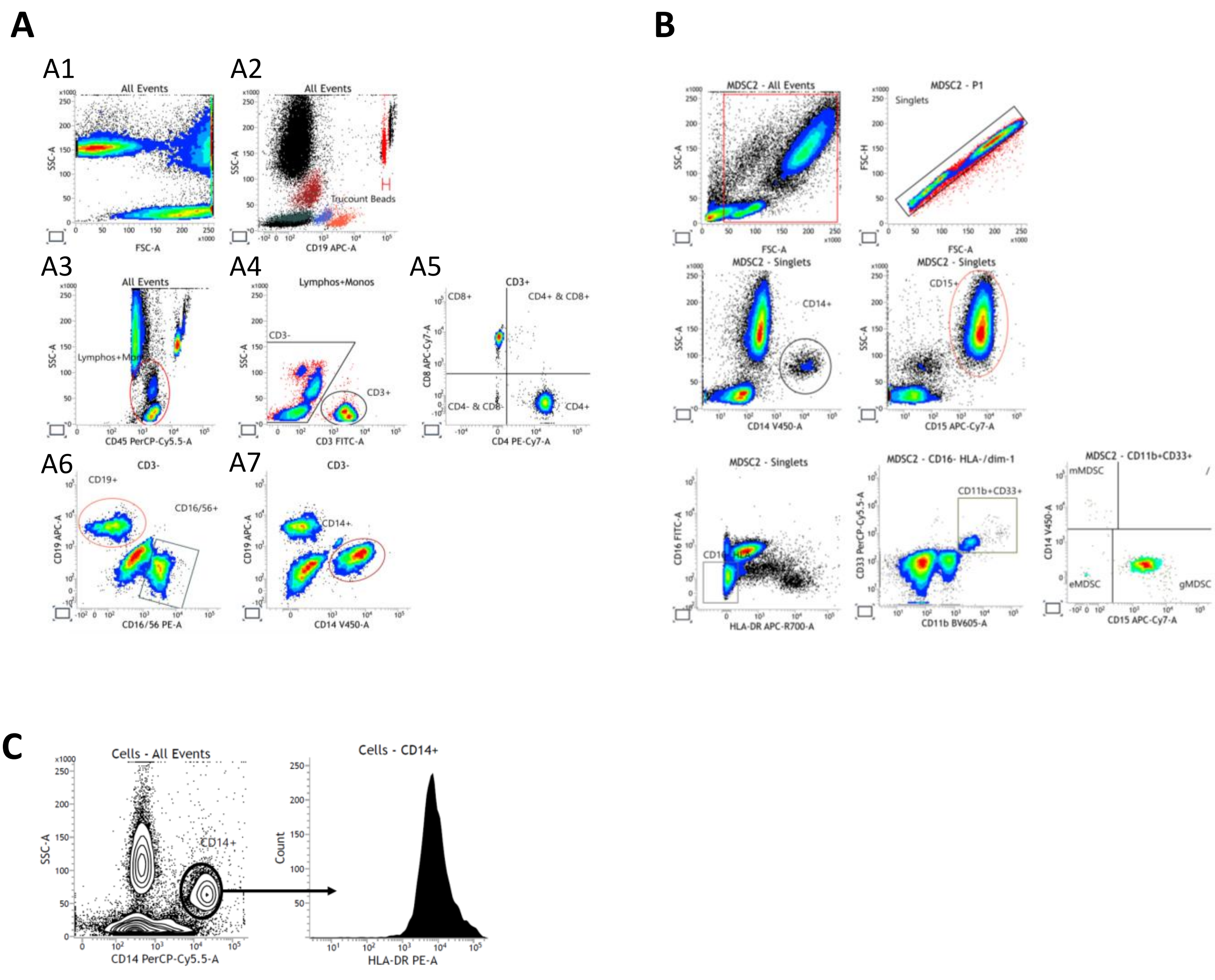

Supplemental Figure 1. **Gating strategies.** (A) Gating strategy for the identification of mononuclear cell populations within the isolated PBMC fraction. Peripheral blood mononuclear cells were stained using a TBNK panel supplemented with an anti-CD14 antibody, and absolute cell counts were determined using Trucount beads. (A1) Exclusion of debris based on forward and side scatter (SSC-A/FSC-A). (A2) Identification of Trucount beads for absolute cell quantification. (A3) Gating of leukocytes based on CD45 expression (Lymphos+Monos). (A4) Separation of CD3$^+$ T cells and CD3$^-$ cells within the lymphocyte/monocyte population. (A5) Subdivision of CD3$^+$ T cells into CD4$^+$ and CD8$^+$ subsets. (A6) Resolution of CD3$^-$ cells into CD19$^+$ B cells and CD16/56$^+$ natural killer cells. (A7) Identification of CD14$^+$ monocytes within the CD3$^-$ population. (B) Identification and quantification of myeloid-derived suppressor cell (MDSC) subsets within the isolated PBMC fraction. Representative gating strategy from one ICU patient. After exclusion of debris (SSC-A/FSC-A) and doublets (FSC-H/FSC-A), CD16$^-$HLA-DR$^-$/dim cells were gated and further selected for CD11b$^+$CD33$^+$ expression. Within this population, granulocytic MDSC (gMDSC; CD15$^+$CD14$^-$), monocytic MDSC (mMDSC; CD14$^+$CD15$^-$), and early-stage MDSC (eMDSC; CD14$^-$CD15$^-$) were distinguished according to the consensus nomenclature of Bronte et al. (for reference see methods section). Subset frequencies vary between individuals; the example shown is from a single representative patient. (C) Gating strategy for quantification of HLA-DR receptor density on CD14$^+$ monocytes (for more detailed description see methods section; Panel C from Siegler et al., PLOS ONE 2021, DOI: https://doi.org/10.1371/journal.pone.0250818; License: CC BY 4.0).
