# Supplementary material for: Genome-wide H3K4me3 profiling of circulating immune cells reveals dynamic epigenetic reprogramming during acute critical COVID-19
Source: Front Immunol. 2026 Aug 20;17:1907275. doi: 10.3389/fimmu.2026.1907275 (PMC13538114; doi:10.3389/fimmu.2026.1907275)
Supplement: Supplementary file 2 [file DataSheet10.pdf]

Suppl. Table 2. Gene Ontology Biological Process (GO BP) enrichment analysis of genes associated with increased H3K4me3 occupancy in ICU patients. GO BP terms enriched among the genes mapped to 706 differentially bound H3K4me3 consensus peaks with promoter association (FDR < 0.05) between ICU (n = 27) and non-ICU patients (n = 19). P-values were calculated by over-representation analysis; adjusted p-values were corrected using the Benjamini-Hochberg method.

GO ID	GO Term	P-value	Adj. P-value	Gene Count
GO:0072672	Neutrophil extravasation	2.17e-6	0.0076	6
GO:0051239	Regulation of multicellular organismal process	3.95e-6	0.0076	130
GO:0060284	Regulation of cell development	4.07e-6	0.0076	36
GO:0045596	Negative regulation of cell differentiation	7.30e-6	0.010	44
GO:0010721	Negative regulation of cell development	1.64e-5	0.017	18
GO:0051093	Negative regulation of developmental process	1.80e-5	0.017	54
GO:0045595	Regulation of cell differentiation	2.68e-5	0.021	79
GO:0045123	Leukocyte extravasation	8.68e-5	0.060	10
GO:2000026	Regulation of multicellular organismal development	1.40e-4	0.087	69
