# Supplementary material for: Genome-wide H3K4me3 profiling of circulating immune cells reveals dynamic epigenetic reprogramming during acute critical COVID-19
Source: Front Immunol. 2026 Aug 20;17:1907275. doi: 10.3389/fimmu.2026.1907275 (PMC13538114; doi:10.3389/fimmu.2026.1907275)
Supplement: Supplementary file 3 [file DataSheet2.pdf]

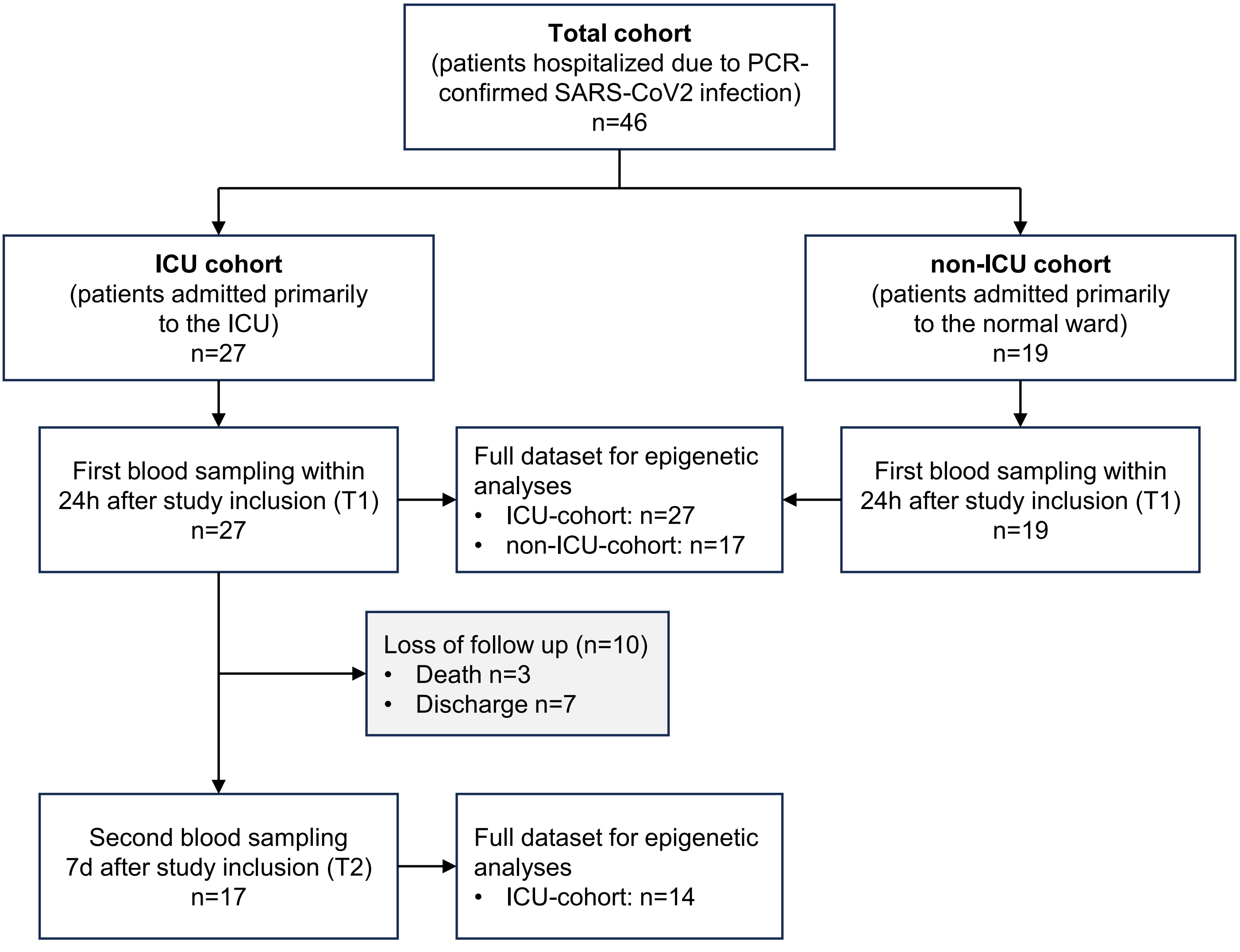

Supplemental Figure 2. **Study cohort.** Abbreviations: ICU intensive care unit, PCR polymerase chain reaction.
