# Supplementary material for: Genome-wide H3K4me3 profiling of circulating immune cells reveals dynamic epigenetic reprogramming during acute critical COVID-19
Source: Front Immunol. 2026 Aug 20;17:1907275. doi: 10.3389/fimmu.2026.1907275 (PMC13538114; doi:10.3389/fimmu.2026.1907275)
Supplement: Supplementary file 4 [file DataSheet3.pdf]

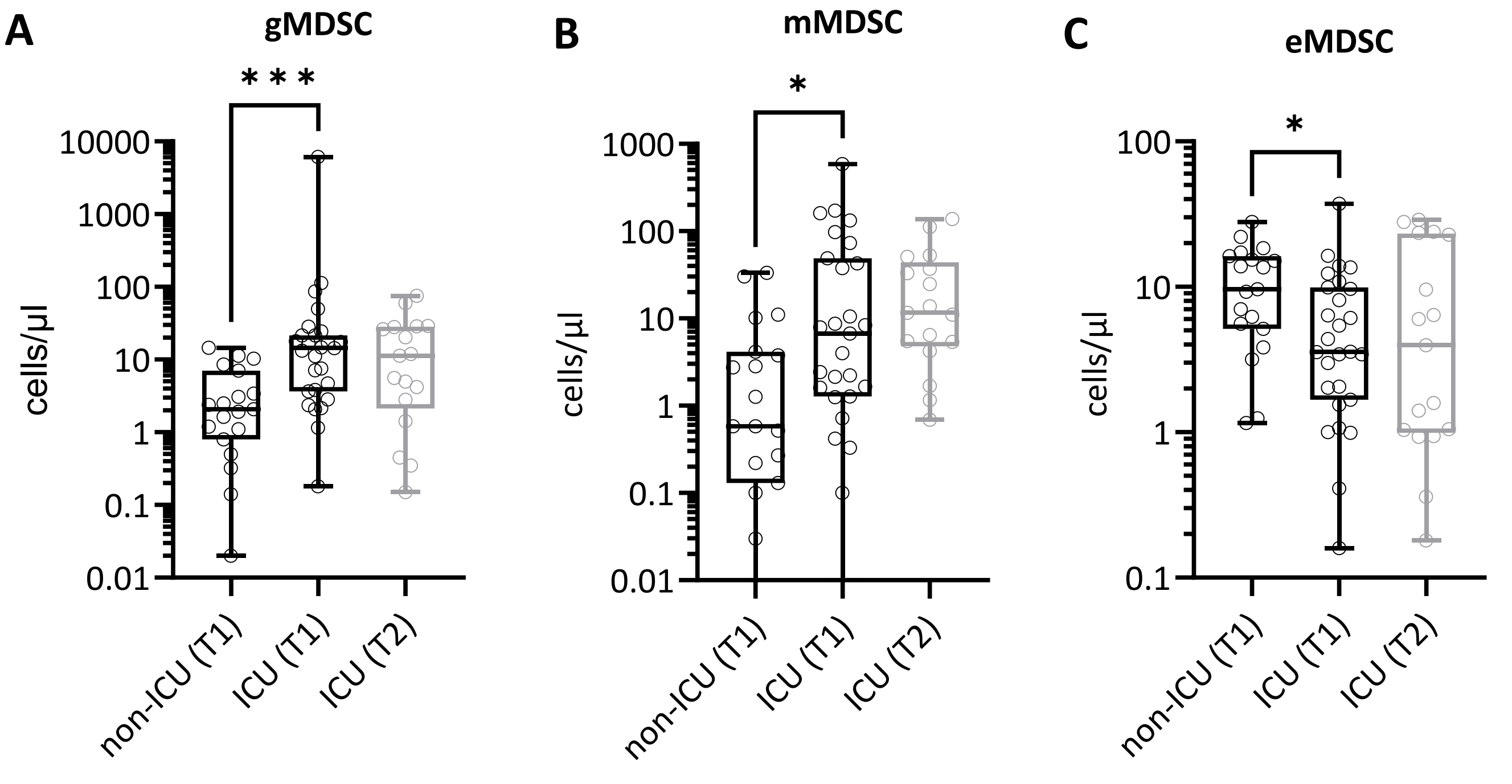

Supplemental Figure 3. **Quantification of myeloid-derived suppressor cell (MDSC) subsets within the isolated PBMC fraction. (A)** Granulocytic MDSC (gMDSC; $CD15^{+}CD14^{-}$), **(B)** monocytic MDSC (mMDSC; $CD14^{+}CD15^{-}$), and **(C)** early-stage MDSC (eMDSC; $CD14^{-}CD15^{-}$) were quantified within the $CD11b^{+}CD33^{+}HLA\text{-}DR^{-}$/dim population of isolated PBMCs by flow cytometry (gating strategy shown in Supplemental Figure 1). Cell concentrations are given as cells/µl for the non-ICU group at hospital admission (non-ICU [T1], n = 19) and for the ICU group at admission (ICU [T1], n = 27) and after seven days (ICU [T2], n = 17). Data are presented as box and whiskers (min to max) with each patient represented as a single data point; note the logarithmic y-axis. Group comparisons between non-ICU and ICU patients at T1 were performed using the two-sided Mann-Whitney U-test (gMDSC, $p < 0.001$; mMDSC, $p = 0.026$; eMDSC, $p = 0.016$). Longitudinal changes within the ICU group between T1 and T2 were assessed using the Wilcoxon signed-rank test and were not statistically significant for any subset.
