# Supplementary material for: Genome-wide H3K4me3 profiling of circulating immune cells reveals dynamic epigenetic reprogramming during acute critical COVID-19
Source: Front Immunol. 2026 Aug 20;17:1907275. doi: 10.3389/fimmu.2026.1907275 (PMC13538114; doi:10.3389/fimmu.2026.1907275)
Supplement: Supplementary file 5 [file DataSheet4.pdf]

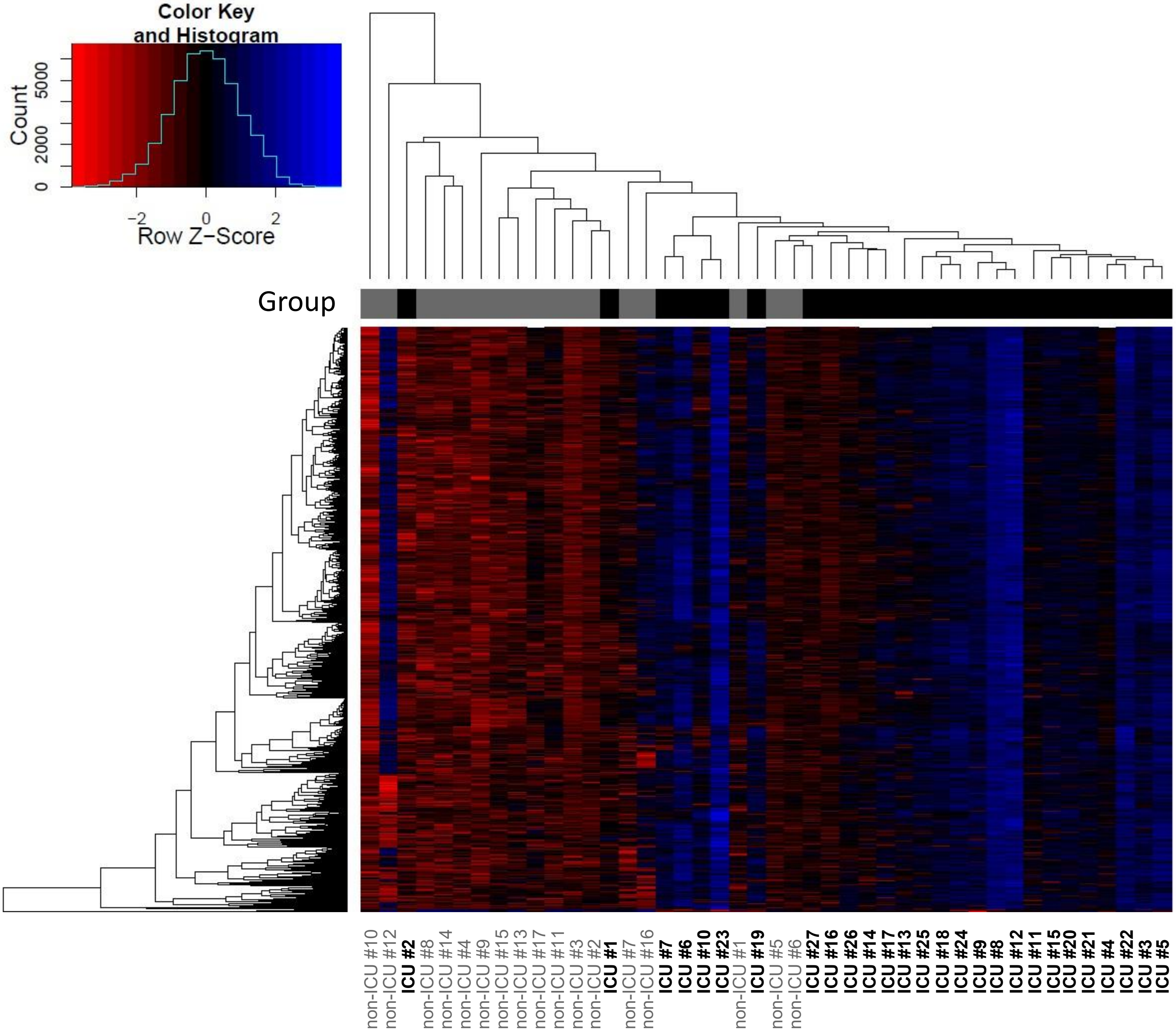

Supplemental Figure 4. **Unsupervised hierarchical clustering of H3K4me3 signal in ICU patients and non-ICU patients**. The heatmap displays normalized H3K4me3 enrichment at genomic regions identified in the DiffBind consensus peak set across all individual patient samples. Samples are color-coded according to group, with ICU patients indicated in black and non-ICU patients in grey. Each heatmap column represents an individual patient sample, and each row represents a consensus peak. H3K4me3 enrichment was scaled on a per-row basis using Z-score normalization to visualize relative differences in enrichment across patients.
