# Supplementary material for: Genome-wide H3K4me3 profiling of circulating immune cells reveals dynamic epigenetic reprogramming during acute critical COVID-19
Source: Front Immunol. 2026 Aug 20;17:1907275. doi: 10.3389/fimmu.2026.1907275 (PMC13538114; doi:10.3389/fimmu.2026.1907275)
Supplement: Supplementary file 8 [file DataSheet7.pdf]

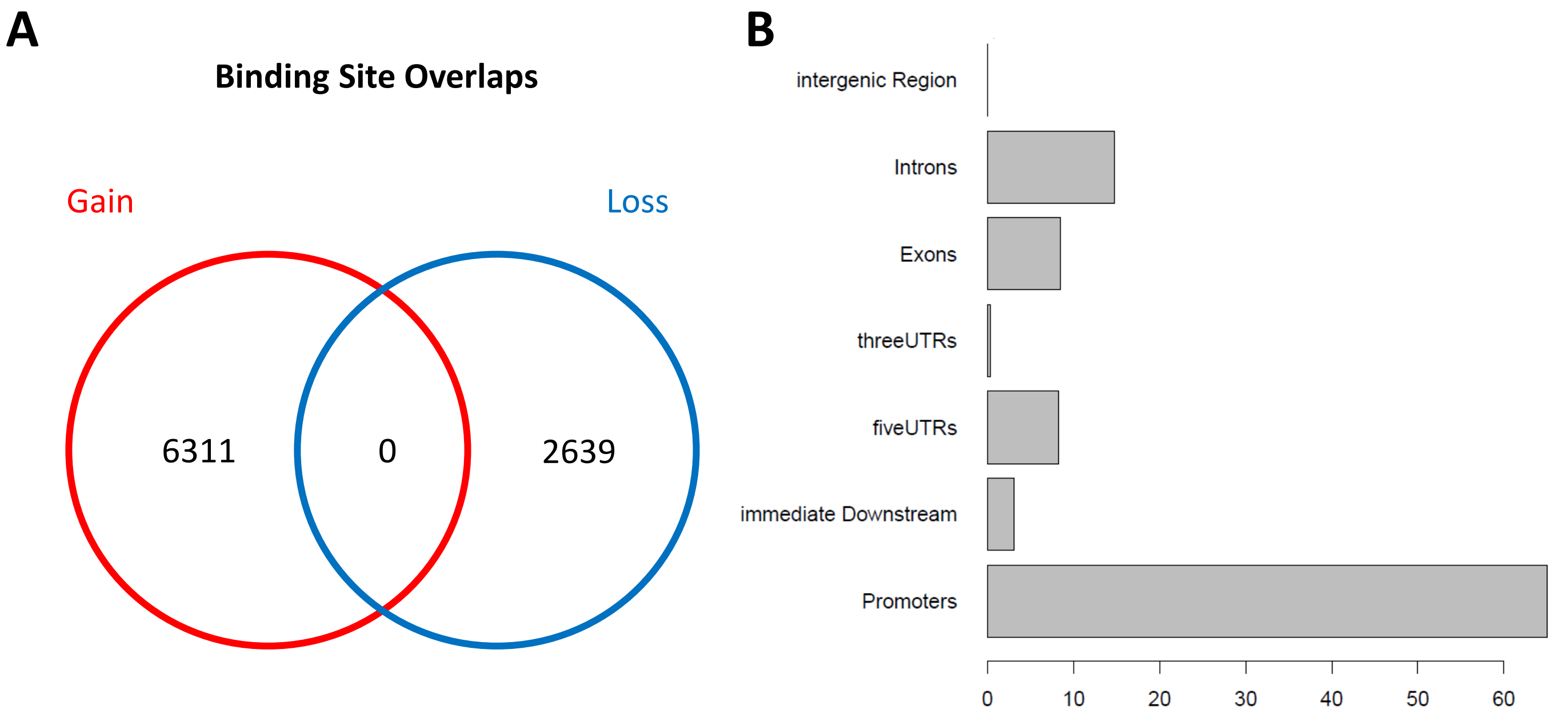

Supplemental Figure 7. **Binding site overlap of differential H3K4me3 binding between ICU patients at T1 and T2 and Genomic distribution of H3K4me3 binding sites. (A)** Venn diagram illustrating the overlap of increased, classified as gain, or reduced, classified as loss, H3K4me3 binding regions. **(B)** Genomic feature distribution of differentially bound H3K4me3 peaks retained after promoter overlap filtering (±1 kb around annotated transcriptional start sites).
