# Supplementary material for: Genome-wide H3K4me3 profiling of circulating immune cells reveals dynamic epigenetic reprogramming during acute critical COVID-19
Source: Front Immunol. 2026 Aug 20;17:1907275. doi: 10.3389/fimmu.2026.1907275 (PMC13538114; doi:10.3389/fimmu.2026.1907275)
Supplement: Supplementary file 9 [file DataSheet8.pdf]

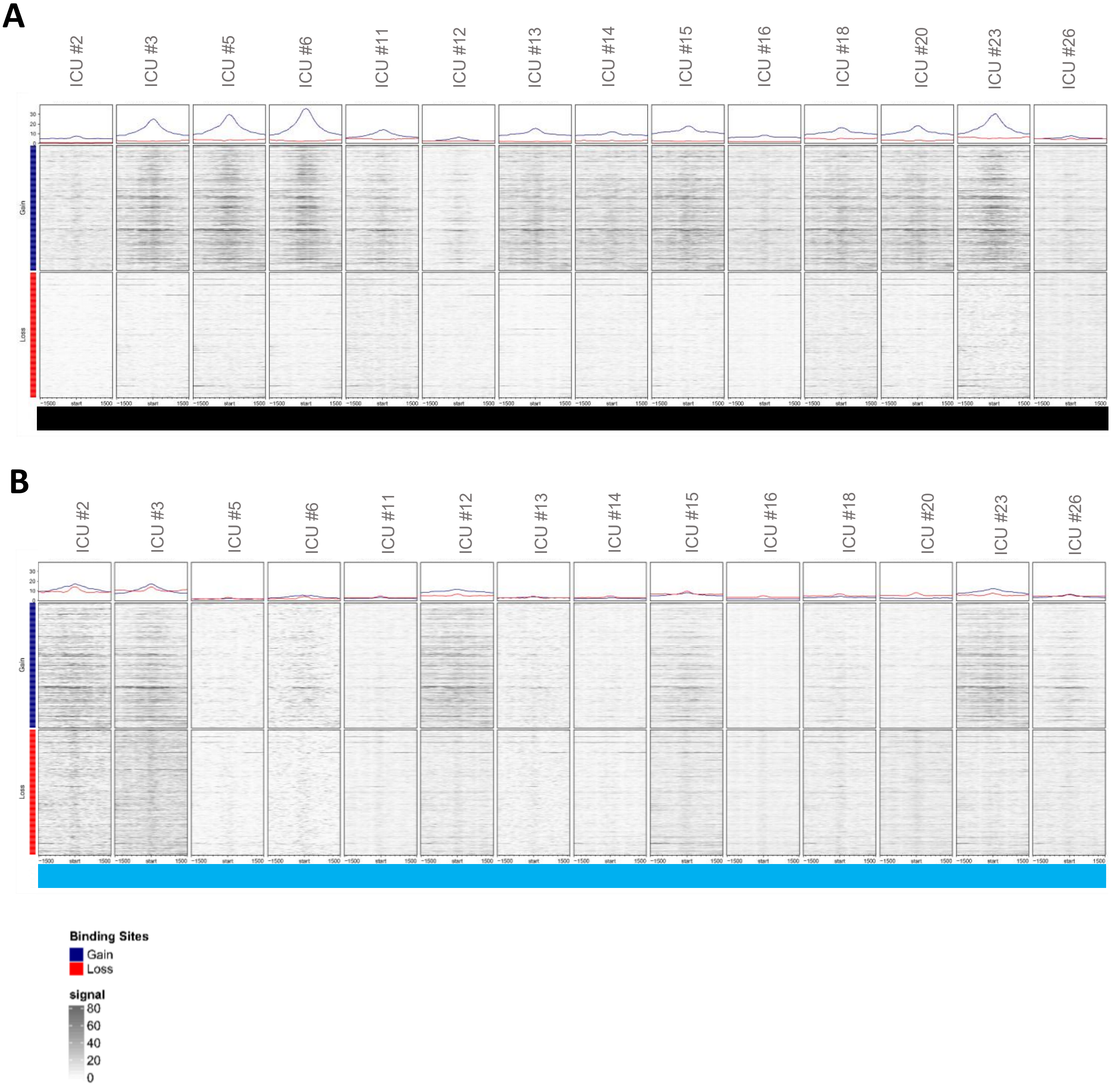

Supplemental Figure 8. **Individual H3K4me3 enrichment in ICU patients at T1 (A) and T2 (B).** Heatmaps and normalized histone modification profiles show H3K4me3 enrichment at genomic regions identified as differentially bound between the two time points by DiffBind analysis. Graphs above the heatmaps summarize the average H3K4me3 enrichment or loss of enrichment across all differentially bound regions. Individual rows in the heatmap represent differentially bound consensus peaks showing either a gain (indicated in blue) or loss (indicated in red) of H3K4me3 enrichment. Differences in signal intensity indicate relative increases or decreases in H3K4me3 enrichment at individual genomic regions. Sample group assignment is indicated by the annotation bar below the profiles, with T1 samples (A) shown in black and T2 samples (B) shown in cyan.
