# Supplementary material for: Genome-wide H3K4me3 profiling of circulating immune cells reveals dynamic epigenetic reprogramming during acute critical COVID-19
Source: Front Immunol. 2026 Aug 20;17:1907275. doi: 10.3389/fimmu.2026.1907275 (PMC13538114; doi:10.3389/fimmu.2026.1907275)
Supplement: Supplementary file 10 [file DataSheet9.pdf]

Suppl. Table 1. Gene Ontology Cellular Component (GO CC) enrichment analysis of genes associated with increased H3K4me3 occupancy in ICU patients. GO CC terms enriched among the genes mapped to 706 differentially bound H3K4me3 consensus peaks with promoter association (FDR < 0.05) between ICU (n = 27) and non-ICU patients (n = 19). P-values were calculated by over-representation analysis; adjusted p-values were corrected using the Benjamini-Hochberg method.

GO ID	GO Term	P-value	Adj. P-value	Gene Count
GO:0042581	Specific granule	6.5e-9	4.34e-6	21
GO:0030141	Secretory granule	3.9e-8	1.30e-5	55
GO:0099503	Secretory vesicle	7.5e-8	1.68e-5	61
GO:0035580	Specific granule lumen	1.5e-7	2.51e-5	12
GO:0034774	Secretory granule lumen	7.1e-7	9.12e-5	27
GO:0031983	Vesicle lumen	9.6e-7	9.12e-5	27
GO:0060205	Cytoplasmic vesicle lumen	8.5e-7	9.12e-5	27
GO:0070820	Tertiary granule	1.13e-6	9.41e-5	18
GO:1904724	Tertiary granule lumen	2.97e-6	2.20e-4	10
GO:0030667	Secretory granule membrane	1.22e-5	8.18e-4	24
GO:0035577	Azurophil granule membrane	3.50e-5	0.0021	9
GO:0005766	Primary lysosomal granule	1.44e-4	0.0074	14
GO:0042582	Azurophil granule	1.44e-4	0.0074	14

GO ID	GO Term	P-value	Adj. P-value	Gene Count
GO:0031225	Anchored component of membrane	3.53e-4	0.017	14
GO:0009986	Cell surface	7.19e-4	0.032	45
GO:0098590	Plasma membrane region	8.09e-4	0.034	55
GO:0031410	Cytoplasmic vesicle	0.0010	0.038	96
GO:0071944	Cell periphery	0.0010	0.038	221
GO:0097708	Intracellular vesicle	0.0011	0.038	96
GO:0031226	Intrinsic component of plasma membrane	0.0025	0.084	70
